# Differential proteomic analysis of replanted *Rehmannia glutinosa* roots by iTRAQ reveals molecular mechanisms for formation of replant disease

**DOI:** 10.1186/s12870-017-1060-0

**Published:** 2017-07-10

**Authors:** Mingjie Li, Yanhui Yang, Fajie Feng, Bao Zhang, Shuqiang Chen, Chuyun Yang, Li Gu, Fengqing Wang, Junyi Zhang, Aiguo Chen, Wenxiong Lin, Xinjian Chen, Zhongyi Zhang

**Affiliations:** 1 College of Crop Sciences, Fujian Agriculture and Forestry University, Fuzhou, China; 2 College of Bioengineering, Henan University of Technology, Zhengzhou, China; 3 Henan Agricultural University, Zhengzhou, China; 4 College of Chemical Engineering, Huaqiao University, Xiamen, China; 5 Key Laboratory of Crop Ecology and Molecular Physiology, Fujian Agriculture and Forestry University, Fuzhou, China

**Keywords:** *R. glutinosa*, Transcriptome, Proteins, iTRAQ, Replant disease, Molecular mechanism

## Abstract

**Background:**

The normal growth of *Rehmannia glutinosa*, a widely used medicinal plant in China, is severely disturbed by replant disease. The formation of replant disease commonly involves interactions among plants, allelochemicals and microbes; however, these relationships remain largely unclear. As a result, no effective measures are currently available to treat replant disease.

**Results:**

In this study, an integrated *R. glutinosa* transcriptome was constructed, from which an *R. glutinosa* protein library was obtained. iTRAQ technology was then used to investigate changes in the proteins in replanted *R. glutinosa* roots, and the proteins that were expressed in response to replant disease were identified. An integrated *R. glutinosa* transcriptome from different developmental stages of replanted and normal-growth *R. glutinosa* produced 65,659 transcripts, which were accurately translated into 47,818 proteins. Using this resource, a set of 189 proteins was found to be significantly differentially expressed between normal-growth and replanted *R. glutinosa*. Of the proteins that were significantly upregulated in replanted *R. glutinosa*, most were related to metabolism, immune responses, ROS generation, programmed cell death, ER stress, and lignin synthesis.

**Conclusions:**

By integrating these key events and the results of previous studies on replant disease formation, a new picture of the damaging mechanisms that cause replant disease stress emerged. Replant disease altered the metabolic balance of *R. glutinosa*, activated immune defence systems, increased levels of ROS and antioxidant enzymes, and initiated the processes of cell death and senescence in replanted *R. glutinosa*. Additionally, lignin deposition in *R. glutinosa* roots that was caused by replanting significantly inhibited tuberous root formation. These key processes provide important insights into the underlying mechanisms leading to the formation of replant disease and also for the subsequent development of new control measures to improve production and quality of replanted plants.

**Electronic supplementary material:**

The online version of this article (doi:10.1186/s12870-017-1060-0) contains supplementary material, which is available to authorized users.

## Background

*Rehmannia glutinosa* Libosch., commonly called Chinese foxglove, is a perennial, herbaceous medicinal plant in the family Scrophulariaceae. This species has been cultivated in China for more than 1000 years and is widely used to treat a variety of health problems without causing side effects [1]. Many pharmaceutically active compounds, including sugars, amino acids, vitamins, iridoids, aucubin, and rehmannin, have been identified in the tuberous roots of *R. glutinosa*, which are the primary medicinal organ [1]. Thus, *R. glutinosa* is highly valued for nutrition and as an herbal medicine in China. However, productivity and quality significantly decline when *R. glutinosa* is planted in a field in which the species was grown in previous years; this decline is commonly described as “replant disease” or “the consecutive monoculture problem”. The problem is not particularly prevalent in *R. glutinosa* but has been reported in various medicinal, vegetable and horticultural plants [2]. Replant disease severely affects the growth and development of *R. glutinosa*, including tuberous root formation, and no effective treatments are currently available.

Researchers generally considered three primary mechanisms for the cause of replant disease: soil nutrient imbalances, shifts in microbial communities towards more pathogenic taxa and allelopathic autotoxicity [3–6]. From preliminary studies, the long-term monoculture of one plant species in a region was thought to lead to a deficiency in certain nutrients that are essential to the plants [3]. However, with the timely supply of these critical elements in practice, the adverse effects caused by replanting did not improve; thus, nutrient deficiencies in soil are not the limiting factor that leads to the formation of replant disease [3]. Plant roots continuously release a series of low-molecular-weight (LMWs, e.g., sugars, amino acids, vitamins, nucleotides and phenolics) and high-molecular-weight (HMWs, e.g., polymerized sugars and proteins) compounds and a subset of secondary metabolites into the rhizosphere as exudates during growth [7]. Accumulation of these compounds can have significant negative effects and contribute to the phenomenon of allelopathic autotoxicity, with the released metabolites acting as autotoxic allelochemicals [8]. Candidate allelochemicals have been widely identified from the rhizosphere of different plants and include phenolics, alkaloids, long-chain fatty acids, terpenoids and flavonoids [9].

In recent studies, root exudates were found to significantly induce the proliferation of rhizosphere microbes and influence microbial communities of the *R. glutinosa* rhizosphere, resulting in the death of replanted plants [10, 11]. Recently, autotoxic allelochemicals derived from the rhizosphere were identified as the most important factors leading to the formation of replant disease, in addition to promoting a shift in microbial communities [12–14]. While plants experiencing replant disease encounter allelochemicals, especially those released from the plants themselves, many physiological and biochemical processes of these plants are seriously impacted and even irreversibly disrupted by allelochemicals. For example, some studies have indicated that allelochemicals can limit the ability of the plants to take up essential ions, solutes and water by inhibiting membrane H^+^-ATPase activity [15–17]. Other reports found that allelochemicals can significantly affect respiration by disturbing oxidative phosphorylation, the normal function of mitochondria, and the ATP synthase activity of the plant [17, 18]. Other reports showed that allelochemicals induce ROS (reactive oxygen species) accumulation and inhibit the antioxidant systems of plants, resulting in membrane lipid peroxidation and impairing the structure and function of the entire cell membrane [19–23]. However, the complete mechanism of how plants suffer from allelochemicals remains largely unknown, particularly at the molecular level.

More recently, some researchers have begun to explore the injury mechanisms of allelochemicals. For example, exogenous ferulic acid and juglone can inhibit the growth of rice seedlings and induce a large number of response genes, from which ROS, calcium signalling, ethylene (ET) and jasmonic acid (JA) may participate plant sensing of allelochemicals [24, 25]. In our previous studies, a set of genes that respond to replanting was identified in roots and leaves of *R. glutinosa* using RNA-seq and DGE (digital gene expression profiling) technology. Functional analysis of these genes suggests that some metabolic pathways, including DNA replication, RNA and protein synthesis, cell division, hormone responses, and chromatin modification, are severely inhibited in replanted *R. glutinosa*. Similarly, the involvement of calcium signalling and ET synthesis in the formation of *R. glutinosa* replant disease was also demonstrated [26, 27]. These results reveal the molecular basis for replant disease formation and provide a better understanding of the consecutive monoculture problem.

mRNA is translated only into proteins that perform cellular functions. Therefore, the important events corresponding to replant disease that are identified at the transcript level must be supplemented with further information at the protein level to reveal more comprehensive mechanisms of the formation of replant disease. iTRAQ (Isobaric Tag for Relative and Absolute Quantification)-based proteomic studies are widely applied in systems that range from stress responses of microorganisms to evaluations of mammalian organelles. An iTRAQ system is used for rapid identification of proteins in a profile and for quantification of differentially expressed proteins [28–32]. Additionally, proteomic studies have been successfully used to detect some key response factors at the molecular level in specialized environments at a low cost. In this study, for a deeper understanding of the mechanisms that lead to the formation of replant disease, transcriptome libraries, including information on expression in different developmental stages and in plants of different status, were constructed. Simultaneously, this information was integrated with previous *R. glutinosa* transcriptomes to create a full transcriptome. Based on this information, the protein profile of replanted *R. glutinosa* roots was identified using iTRAQ at critical damaging stages of replant disease. Proteins that were differentially expressed between normal-growth and replanted *R. glutinosa* were analysed in detail by integrating their functional annotations from different datasets. This analysis developed the complete molecular process by which the plants sensed and responded to replant stress. The results highlighted the causal relationship between the disruption of metabolic balance and the generation of autotoxic allelochemicals and revealed the roles of molecular events triggered by the immune defence response in replanted *R. glutinosa*.

## Methods

### Design and construction of the isolation plots and planting of plant material

**Fig. 1:**
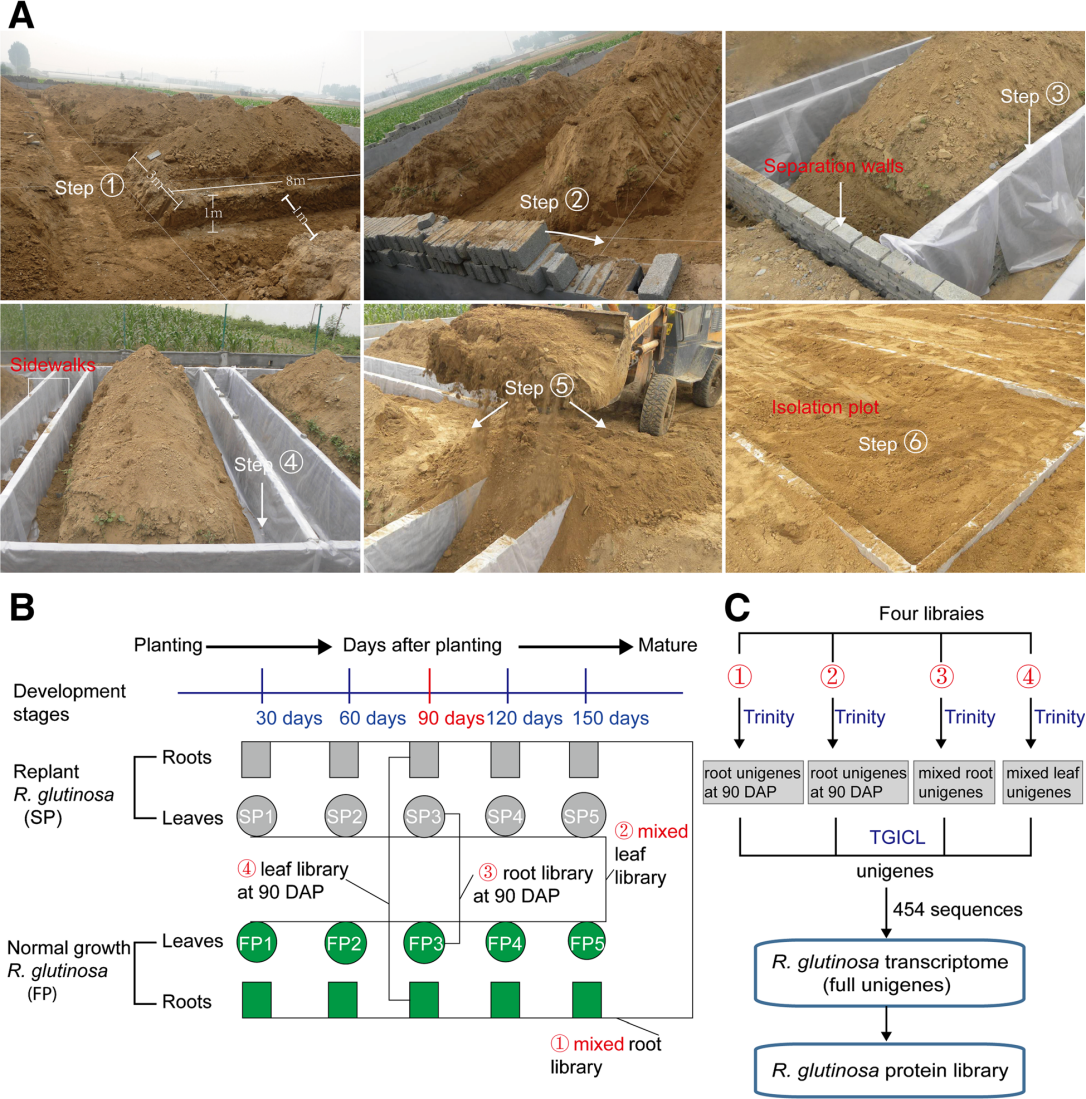
**a** The construction of the isolation plots for *R. glutinosa* plants. The soil was first removed from the walkways between any two plots (left and right sides), to a depth greater than 1 m, as was soil from the other two sides of each plot. The soil that was dug from these locations was moved away from the plots. The separation walls were then built on the four sides of each plot using brick (Step 2), and the separation walls were then evenly covered with impermeable membranes (Step 3) that were placed tightly against the walls (Step 4). After the separation walls of each plot were built, the soil was backfilled into the plots and walkways (Step 5). Finally, the surfaces of the plots were levelled and made consistent with each other (Step 6). **b** Construction of *R. glutinosa* transcriptome libraries and **c** assembly of unigene sequences generated from different libraries

The experimental site for this study was chosen at the Wen Agricultural Institute, Jiaozuo City, Henan Province, China. No specific permissions were required for this field experiment or the related activities. This county is well known as the geo-authentic zone for *R. glutinosa* cultivation, as *R. glutinosa* grown there has higher medicinal quality than that from other regions of China [1, 33]. An experimental region that was never planted with *R. glutinosa* was separated from the adjacent region using wire mesh to prevent potential interference caused by other plants. A total of 6 plots of 8 m × 3 m each, with 1-m-deep separation walls, were constructed in this experimental field. Simultaneously, 2-m-wide walkways were placed between adjacent isolation plots to avoid the influence of different treatments on each other (Fig. 1a).

A group of *R. glutinosa* (Wen 85–5) seedlings was grown in the isolation plots in which the same cultivar was planted the previous year. Another group of seedlings was grown in the isolation plots in which *R. glutinosa* had never been planted. For a more convenient description of these groups, the former was the normal-growth *R. glutinosa* or first-year planting (FP) group, and the latter was the replanted *R. glutinosa* or second-year planting (SP) group. *R. glutinosa* plants were collected from the GAP Institute of Fujian Agriculture and Forestry University. Because this species has been widely grown and is not an endangered or protected species in China, no specific permission was required for collecting *R. glutinosa* samples. Six plots were planted with FP and SP plants, of which 3 were used for FP and 3 for SP. These plots were termed isolation plots, and the soils were identical in the different isolation plots. These soils were mainly composed of sandy loam, which is considered favourable for growing *R. glutinosa*. Before our experiment, analysis of the nutrient content was conducted on the soils of the isolation plots. This analysis showed that the soils used for FP had an organic matter content of 13.02 g·kg^−1^, a pH of 7.15, total nitrogen content of 0.58 g·kg^−1^, available nitrogen content of 75.31 mg·kg^−1^, effective phosphorus content of 31.30 mg·kg^−1^ and available potassium content of 255.24 mg·kg^−1^. The soils used for SP had an organic matter content of 12.60 g·kg^−1^, a pH of 7.05, total nitrogen content of 0.58 g·kg^−1^, available nitrogen content of 79.66 mg·kg^−1^, effective phosphorus content of 23.90 mg·kg^−1^ and available potassium content of 169.23 mg·kg^−1^. During the growth and development of *R. glutinosa* plants, the same field management was performed for SP and FP.

### Measurement of biomass; root and SOD activities; MDA, ET, and H_2_O_2_ content; and Ca^2+^ density

Ten SP and FP plants were randomly sampled 30 days after planting (DAP) and at 30 day intervals thereafter. The sampled plants were carefully washed, placed in a 70 °C oven, and dried until constant weight for the dry matter determination of both above- and below-ground biomasses. Root activity was determined by the TTC (triphenyl tetrazolium chloride) method [34]. TTC can be reduced to formazan by dehydrogenase enzymes, and root activity can thus be expressed by the decrease in TTC absorbance at 485 nm by the following formula: root activity = amount of TTC reduction (μg)/fresh root weight (g) × time (h). SOD (superoxide dismutase) activities were measured by the NBT method [35]. Fresh roots were cut into small pieces (1–2 cm) and ground to a powder with a mortar on ice. Raw powders were homogenized using 5 ml of precooled 100 mM potassium phosphate buffer (pH = 7.8) containing 1 mM EDTA, 5 mM dithiothreitol and 5% (*w*/*v*) polyvinylpyrrolidone. The homogenate was centrifuged at 13000×*g* for 20 min at 4 °C, and the supernatant was used for SOD enzyme assays. One unit of SOD activity was defined as the amount of enzyme that inhibited the NBT photoreduction reaction by 50% at 560 nm. The MDA content was measured by the thiobarbituric acid (TBA) method [36]. In brief, a total of 2 ml of the supernatant was added to 2 ml of 0.6% (*w*/*v*) TBA solution dissolved in 5% (*v*/v) trichloroacetic acid. The mixtures were heated in boiling water for 10 min and cooled to allow the sediment to flocculate. The absorbances of the supernatant at 450 nm and 532 nm were measured and subtracted from the absorbance at 600 nm. The MDA content was defined as the amount per gram of fresh roots (nmol•g^−1^ FW).

Roots of SP and FP at the root expansion stage (90 DAP) were sampled to determine ET and H_2_O_2_ content and Ca^2+^ level. ET levels were determined using a one-step double-antibody sandwich enzyme-linked immunosorbent assay (ELISA). Briefly, 1 g of roots was ground in 0.01 M PBS buffer (pH = 7.4) with an ice-cooled mortar and centrifuged at 2500×*g* for 15 min at 4 °C to obtain supernatant for the ELISA. Coating with purified ET antibody, incubating, washing, adding ET antibody coupled with HRP, incubating, washing, colouring and assaying were then conducted as described by the manufacturer of the Plant ET ELISA Kit (Shanghai Kanu Biological Technology, Shanghai, China). The ET content was measured at 450 nm using a microplate reader. The H_2_O_2_ content was determined by a modified Ti(IV)–H_2_O_2_ method [37]. In short, 1 g of roots was homogenized in 5 ml precooled acetone with a small amount of quartz sand. Extracts were centrifuged for 10 min at 3000×*g*, and 0.5 ml of 20% titanic tetrachloride in concentrated HCl was added to each supernatant, followed by the addition of 3.5 ml of NH_4_OH to precipitate the peroxide-titanium complex. The mixture was centrifuged at 3000×*g* for 10 min, and the supernatant was discarded. After the precipitate was washed 5 times with acetone, it was solubilized with 5 ml of 2 M H_2_SO_4_. The absorbance of the final solution was measured at 415 nm. A standard curve was used to determine the concentration of H_2_O_2_ in the extract. The Ca^2+^ density was determined by calcium fluorescent dye. Fresh root tips were rapidly washed and incubated in 50-μM calcium fluorescent dye (Fluo-3/AM, Life) for 2 h at 4 °C in the dark, and some samples were incubated in free Fluo-3/AM under the same conditions as a background control. Samples were then washed again and incubated in free Fluo-3/AM buffer at 20 °C. Samples were subsequently made into temporary imprints, and tomography scanning was carried out by confocal fluorescence microscopy (Olympus) to observe Ca^2+^ density in roots. In addition, roots harvested 90 DAP were used to prepare homogenate for determination of catalase (CAT) and peroxidase (POD) activity according to the methods used to prepare homogenate for testing SOD activity. POD activity was measured by monitoring the change in absorbance at 420 nm within certain intervals upon enzyme-catalysed oxidation of the substrate (guaiacol) [38]. CAT activity was expressed as absorbance change per min per g fresh roots [39]. Measurement of all physiological and biomass indexes were conducted in three independent biological samples. Statistical analysis was performed using Statistical Package for Social Sciences software (version 19.0). Significant differences were analysed using Fisher’s least significant difference (LSD) test and one-way ANOVAs.

### Transcriptome assembly and construction of protein reference library

Root and leaf samples at different stages were collected from SP and FP at 30-day intervals. Ten root samples from SP (5 stages) and FP (5 stages) were mixed to construct a root transcriptome library; 10 leaf samples were used for a leaf transcriptome library (Fig. 1b). Raw reads were generated with an Illumina HiSeq™ 2500 system, and those carrying only a 3′ adapter sequence were removed. The de novo assemblies for root and leaf raw reads were performed using Trinity software. The output of this analysis was two sets of unigenes from mixed root and mixed leaf samples from all developmental stages (Fig. 1b, c). The mixed unigene sets from root and leaf samples in the present study were integrated with unigenes generated from previous studies and other platforms. First, mixed unigenes from two libraries from roots (mixed from FP and SP at 90 DAP) and leaves (mixed from FP and SP at 90 DAP) from our previous study were reassembled into root and leaf unigenes, respectively (Fig. 1b, c) [26, 27]. Second, four sets of unigenes were integrated with sequences generated from 454 platforms by Sun et al. [40] into the full *R. glutinosa* transcriptome (Fig. 1c).

To predict the coding regions of transcriptome sequences (CDSs) and obtain the *R. glutinosa* protein reference database, unigenes in the entire transcriptome were first aligned to protein databases in the priority order of Nr, Swiss-Prot, KEGG and COG, using blastx (e-value < 10^−5^). First, unigenes were aligned to the higher-priority protein databases. If an alignment for a unigene was found in a higher priority database, that unigene would not be aligned to a lower priority database; otherwise, the unigene was aligned with the next protein database until all alignments were finished. Second, the proteins with the highest ranks in the blast results were used to determine the coding regions of unigenes, and the coding regions were then translated into amino sequences with the standard codon table. Third, unigenes that were not aligned to any database were scanned by ESTScan [41] to obtain the nucleotide (5′-3′) and amino acid sequences of the predicted coding regions. Finally, the protein sequences translated from CDSs from blast results and predicted by ESTScan were combined into *R. glutinosa* protein libraries.

### Protein extraction and digestion

At the root expansion stage (90 DAP), five FP roots with two biological replicates were randomly collected from two of the three FP separation plots, and SP roots were sampled in the same way. Total proteins were extracted from two replicates of FP and SP roots using the TCA procedure. Briefly, the samples were ground into fine powder in liquid nitrogen with a mortar and pestle. After approximately 5 volumes of TCA/acetone (1:9) were added to the powder and mixed by vortexing, the mixture was placed at −20 °C for 4 h and then centrifuged at 6000×*g* for 40 min at 4 °C, and the supernatant was discarded. The precipitate was washed three times with precooled acetone and air dried. The powder was collected and dissolved in 30 volumes of SDT buffer (4% SDS, 100 mM DTT and 150 mM Tris-HCl, pH = 8.0) per 20–30 mg and boiled for 5 min. The lysate was sonicated, boiled for 15 min, and then centrifuged at 14000×*g* for 40 min. The supernatant was filtered with 0.22-μm filters and quantified using the BCA method.

A total of 300 μg of protein from each sample was dissolved in 4% SDS, 100 mM DTT and 150 mM Tris-HCl (pH = 8.0) and further washed using 8 M urea with 150 mM Tris-HCl (pH = 8.0) by repeated ultrafiltration (Microcon units, 10 kD) to remove detergent, DTT and LMWs. After 100 μl of iodoacetamide (100 mM IAA in UA buffer) was added to block reduced cysteine residues, the samples were incubated for 30 min in darkness. Subsequently, the filters were washed three times with 100 μl UA buffer and twice with 100 μl dissolution (DS) buffer. Finally, the protein suspensions were digested with 4 μg trypsin in 40 μl DS buffer overnight at 37 °C, and the resulting peptides were desalted on C18 cartridges.

### Labelling and peptide fractionation

A total of 100 μg of peptide mixture for each sample was labelled with iTRAQ reagents according to the manufacturer’s instructions (iTRAQ Reagent Multiplex Kit, AB SCIEX, Framingham, MA, USA). Two biological replicates from SP were labelled with 116 and 121 and two from FP with 115 and 119. The labelling reactions were processed for 1 h at room temperature. The labelled peptides were fractionated by SCX chromatography using the AKTA Purifier system (GE Healthcare, Fairfield, CT, USA). The dried peptide mixture was reconstituted and acidified with buffer A (10 mM KH_2_PO_4_ in 25% ACN, pH 3.0) and loaded onto a PolySULFOETHYL 4.6 × 100 mm column (5 μm, 200 Å, PolyLC Inc., Maryland, USA). The peptides were eluted at a flow rate of 1 ml/min with a gradient of 0–8% buffer B (500 mM KCl, 10 mM KH_2_PO_4_ in 25% ACN, pH = 3.0) for 22 min, 8–52% buffer B during minutes 22–47, 52–100% buffer B during minutes 47–50, and 100% buffer B during minutes 50–58, and buffer B was reset to 0% after 58 min. The elution was monitored by absorbance at 214 nm, and a total of 30 fractions were obtained; these were further combined into 10 fractions. The collected fractions were lyophilized by a vacuum concentrator and descaled using a C18 cartridge (Sigma, Gillingham, UK).

### Reverse-phase nanoflow HPLC and tandem mass spectrometry

SCX fractions were dissolved in buffer A (0.1% formic acid) and loaded onto a reversed-phase trap column (Thermo Scientific Acclaim PepMap100, 100 μm × 2 cm, nanoViper C18). Peptides were separated with a linear gradient of buffer B (84% acetonitrile and 0.1% formic acid) at a flow rate of 300 nl/min. The linear phase-gradient was set as follows: 0–35% buffer B for 50 min, 35–100% buffer B for 5 min, hold in 100% buffer B for 5 min. A Q-Exactive mass spectrometer (Thermo Finnigan, San Jose, CA, USA) was used to acquire data in the positive ion mode, with a selected mass range of 300–1800 mass/charge (*m*/*z*). Survey scans were acquired at a resolution of 70,000 at 200 *m*/*z*; resolution for HCD spectra was set to 17,500 at 200 *m*/*z*, and isolation width was 2 *m*/*z*. MS/MS data were acquired using a data-dependent top10 method, dynamically choosing the most abundant precursor ions. Normalized collision energy was 30 eV, and the underfill ratio, which specifies the minimum percentage of the target value likely to be reached at the maximum fill time, was defined as 0.1%.

### Protein identification and quantification

MS/MS spectra were searched using the MASCOT engine (Matrix Science, London, UK; version 2.2) embedded into Proteome Discoverer 1.4 against the *R. glutinosa* proteome database. The following parameters were set: trypsin as the enzyme, monoisotopic mass, fragment tolerance at 0.1 Da, peptide mass tolerance at ±20 ppm and allowing up to two missed cleavages. Fixed modifications were defined as iTRAQ labelling and carbamidomethylation of cysteine; oxidation of methionine was specified as a variable modification. The decoy database pattern was set as a reversed version of the target database. False discovery rate (FDR) of peptide identification was set as FDR ≤ 0.001. Protein identifications were supported by a minimum of one unique peptide identification.

The values of ion peak intensities for peptides were extracted using Proteome Discover 1.3, and the signal intensity was normalized through the median value of each label. The quantitative results for peptides were ratios of the signal intensity values between the other labels and the reference sample label. The quantitative result for each protein was the median of the quantitative results for the peptides identified. To avoid the discrepancies in sample loading volumes caused by human error, the final quantitative results for proteins were further normalized using the median ratio of each label. The quantitative result for a protein was the median of the corresponding quantitative results for the identified peptide. To identify the proteins that were significantly differentially expressed in the two groups of samples, the threshold value for the down-regulated proteins was 0.70-fold and that for the upregulated proteins was 1.30-fold, with *p*-values less than 0.05 that were calculated based on significance A methods [42].

### qRT-PCR analysis

For qRT-PCR, 5 μg of RNase-free DNase I-treated RNA was processed with M-MLV reverse transcriptase (Takara Bio Inc., Japan) according to the manufacturer’s instructions. Relevant PCR primers directed against a selection of differentially transcribed sequences identified by iTRAQ (Additional file 1) were designed using Beacon Designer 8.0 software (Premier Biosoft International, Palo Alto, CA, USA). A fragment of the gene encoding 18S rRNA was used as a reference. PCR was performed with a Bio-Rad iQ5 instrument (Bio-Rad, Hercules, CA, USA), using SYBR Green to detect transcript abundance. Each 25-μL reaction contained 0.5 μM of each primer and approximately 0.5 U of enzymes, cDNA and SYBR Green. Negative control reactions contained no cDNA. Five-fold dilutions of the cDNA templates were tested under conditions identical to those used for the samples being tested. The PCR regime included an initial denaturing step (95 °C/10 s), followed by 40 cycles at 95 °C/5 s, 60 °C/10 s, and 72 °C/15 s and a final stage at 55 °C to 95 °C to determine dissociation curves of the amplified products. All reactions were replicated at least three times. The data were analysed using Bio-Rad iQ5 Optical System Software v2.1. The relative transcript level of each gene was calculated using the 2^-△△CT^ method, and the data were normalized based on the 18S rRNA CT values [43].

## Results

### Comparisons of morphological characteristics between normal-growth and replanted *R. glutinosa*

**Fig. 2:**
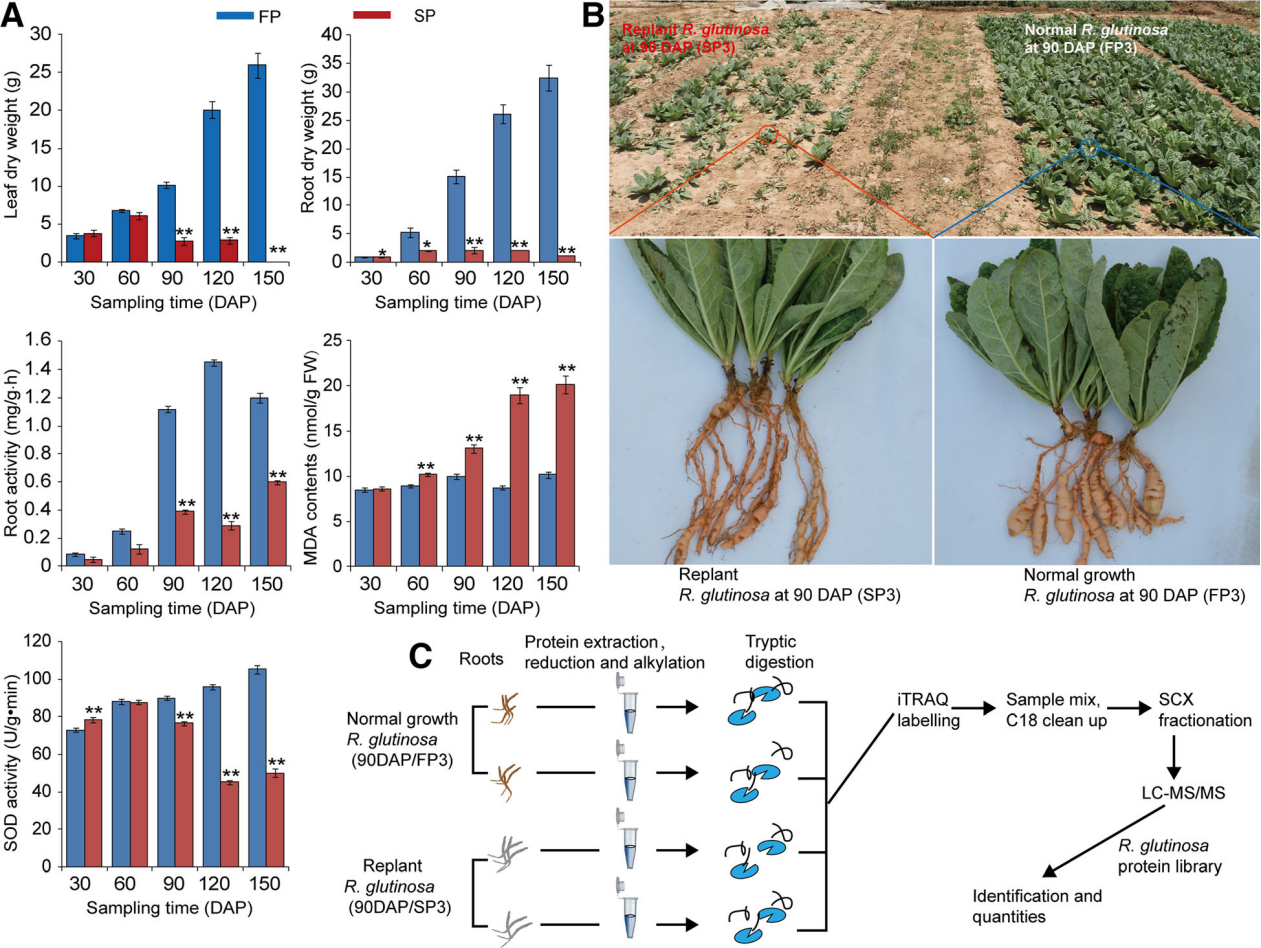
**a** Effects of replant disease on the biomass and related physiological indexes of *R. glutinosa*. **b** Comparison of the appearance of replanted and normal-growth *R. glutinosa* at the most critical harmful stage (90 DAP) caused by replanting and (**c**) an overview of the iTRAQ experimental protocol. Error bars represent standard deviations (SD). * and ** represent significant differences of tested indexes between replanted and normal-growth *R. glutinosa* at *p* < 0.05 and *p* < 0.01, respectively, based on LSD test

The exposure of *R. glutinosa* to replant disease stress resulted in severe inhibition of the growth and development of the plants, as indicated by the decreased below- and above-ground biomass in replanted *R. glutinosa* compared with normal-growth plants. However, the dry weights of the below- and above-ground parts of replanted *R. glutinosa* exhibited inconsistent changes at 30–60 DAP; at that time, the below-ground biomass had begun to show inhibition in replanted *R. glutinosa*, but the above-ground biomass had not. Consistent with the biomass changes of below-ground *R. glutinosa*, the root activity was significantly decreased during this stage in replanted *R. glutinosa* (Fig. 2a). Furthermore, SOD activities were similar at 30 and 60 DAP in replanted and normal-growth *R. glutinosa* plants, but after 90 DAP, SOD activity in replanted *R. glutinosa* decreased. In addition, higher MDA content was detected in replanted *R. glutinosa* at different stages (Fig. 2a). These results indicated that the stage most vulnerable to damage caused by replant disease was the root expansion stage (90 DAP). At that time, SP showed clear damage characteristic of replant disease compared with FP, which agreed with the previous study in *R. glutinosa* (Fig. 2a, b) [26, 27].

### Construction of *R. glutinosa* transcriptome and proteome reference libraries

To obtain a full-length of cDNA transcript and identify proteins responding to replant disease in *R. glutinosa*, the root and leaf transcriptome libraries from normal-growth and replanted *R. glutinosa* at different developmental stages were constructed using an Illumina platform (Fig. 1b). The mixed root and mixed leaf libraries yielded 71,922 and 54,722 unigenes (mixed unigenes), respectively, using Trinity software in the present study (Fig. 1c; Additional file 2). Simultaneously, from a previous study, root and leaf libraries from 90-DAP *R. glutinosa* were reassembled to produce 46,207 and 46,831 unigenes (unigenes at root expansion stage), respectively (Fig. 1b; Additional file 2) [26, 27]. The four sets of unigenes generated from the different libraries and the transcripts from Sun et al. [40] generated by the 454 GS FLX Titanium platform were merged to form the entire *R. glutinosa* transcriptome (full unigenes) using TGICL software (Fig. 1c; Additional file 2). A final set of 66,906 entries was obtained from the *R. glutinosa* transcriptome, with a mean length of 868 bp and an N50 length of 1412 bp, representing 58.7 Mbp of sequence (Additional files 2, 3). These full unigene sequences were carefully inspected and translated into protein sequences. This procedure generated 47,818 proteins entries with lengths ranging from 100 to 3500 amino acids (Additional file 4).

To determine the functional distribution of the proteins derived from the transcriptome, a functional annotation was executed using the Nr, GO, KEGG and COG databases. Of the sequences, 67.8% had significant similarity with entries in the Nr, 40.4% with entries in the KEGG, 51.2% with entries in the GO and 45.6% with entries in the Swiss-Prot protein database (Additional file 5), based on blastx comparisons of the protein sequences using a cut-off e-value of 10^−5^. The GO-based analysis identified genes in the groups “biological processes”, “cellular components” and “molecular function”, which included 46 total categories (Additional file 6). The COG-based annotation classified the putative gene products into at least 25 families (Additional file 7). Finally, the analysis based on KEGG identified genes with involvement in 129 metabolic or signalling pathways (Additional file 8).

### Identification of differentially expressed proteins (DEPs) between normal-growth and replanted *R. glutinosa* and analysis of their functions

To further elucidate the molecular mechanisms underlying the formation of replant disease at the proteins level, iTRAQ was used to identify DEPs between normal-growth and replanted *R. glutinosa* in two biological replicates (Fig. 2c). A total of 17, 479 high-quality peptides were obtained using the *R. glutinosa* protein database (Additional file 9). After merging the two repeats, 4146 non-redundant proteins were identified and quantified (Additional file 10). To define proteins responsive to replant disease, significantly differentially expressed proteins (DEPs) were identified using a 95% confidence level and a cut-off value of 1.30-fold for upregulated and 0.70-fold for down-regulated proteins. This analysis revealed 189 proteins that were differentially expressed in normal-growth compared with replanted *R. glutinosa*. Of these proteins, the abundance of 181 increased and 8 decreased in the replanted *R. glutinosa* library (Additional file 11).

**Fig. 3:**
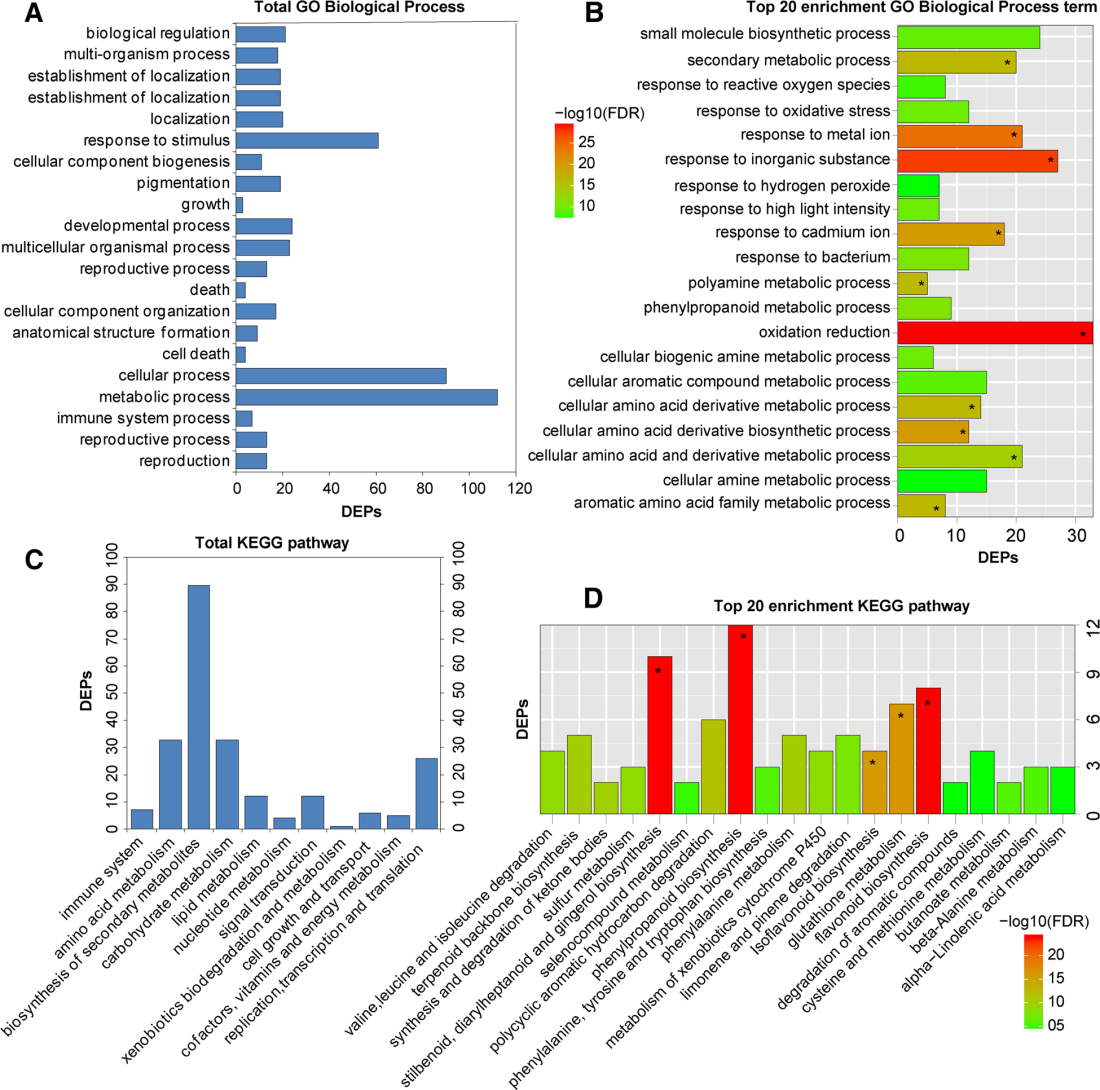
Functional categories of DEPs in replanted *R. glutinosa* compared with normal-growth plants. **a** Total GO biological process categories and **b** GO biological process categories with the top 20 enriched terms. **c** Total KEGG and **d** the top 20 enriched KEGG pathways. * Significantly enriched GO and KEGG categories

**Fig. 4:**
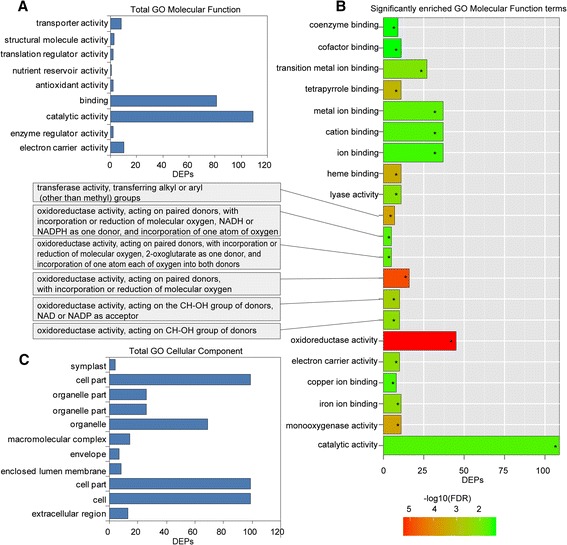
Cellular component and molecular function categories in GO analysis of differentially expressed proteins (DEPs). **a** Total cellular component categories, **b** total GO molecular function categories and (**c**) significantly enriched molecular function categories. * Significantly enriched GO categories

GO and KEGG analyses for the DEPs were also conducted to explore the possible roles of these proteins in replant disease. Among the 189 DEPs, 153 were subcategorized into 41 hierarchically structured GO classes, including 21 biological processes, 11 cellular components, and 9 molecular functions (Figs. 3, 4). In biological processes, the most common categories were metabolic process, cellular process and response to stimulus (Fig. 3a). Catalytic activity and binding were most the common categories in molecular function, whereas in the cellular component class, cell and cell part were most the frequent categories (Fig. 4a, c). Additionally, enrichment analysis for all GO terms indicated that 10 terms in biological processes were significantly enriched based on corrected *p*-values (FDR) <0.05, including oxidation-reduction, response to inorganic substance, response to metal ion, cellular amino acid derivative biosynthetic process, response to cadmium ion, polyamine metabolic process, aromatic amino acid family metabolic process, etc. (Fig. 3b). In molecular functions, a total of 21 terms were enriched, including oxidoreductase activity, monooxygenase activity, haeme binding, and electron carrier activity, among others (Fig. 4b). However, no cellular component terms were significantly enriched.

To investigate which biological pathways were active in replanted *R. glutinosa*, 189 DEPs were classified into 88 KEGG pathways (Additional file 12), and these proteins primarily participated in metabolism, plant-pathogen interaction, and signal transduction (Fig. 3c, d). Within the metabolism category, proteins related to the metabolism of terpenoids, phenylalanine, amino acids, glucose and fatty acids were significantly more abundant in replanted *R. glutinosa*, with proteins involved in calcium and hormone signalling also more abundant (Additional file 12). Additionally, the most active pathways in replanted *R. glutinosa* involved plant-pathogen interaction and RNA transport. KEGG-enriched analyses showed that biosynthetic pathways were significantly enriched, including those for stilbenoids, diarylheptanoids, gingerol, phenylpropanoids, flavonoids, etc., based on corrected *p*-values (FDR) <0.05 (Fig. 3d).

### Comprehensive analysis of DEPs in normal-growth compared with replanted *R. glutinosa*

**Fig. 5:**
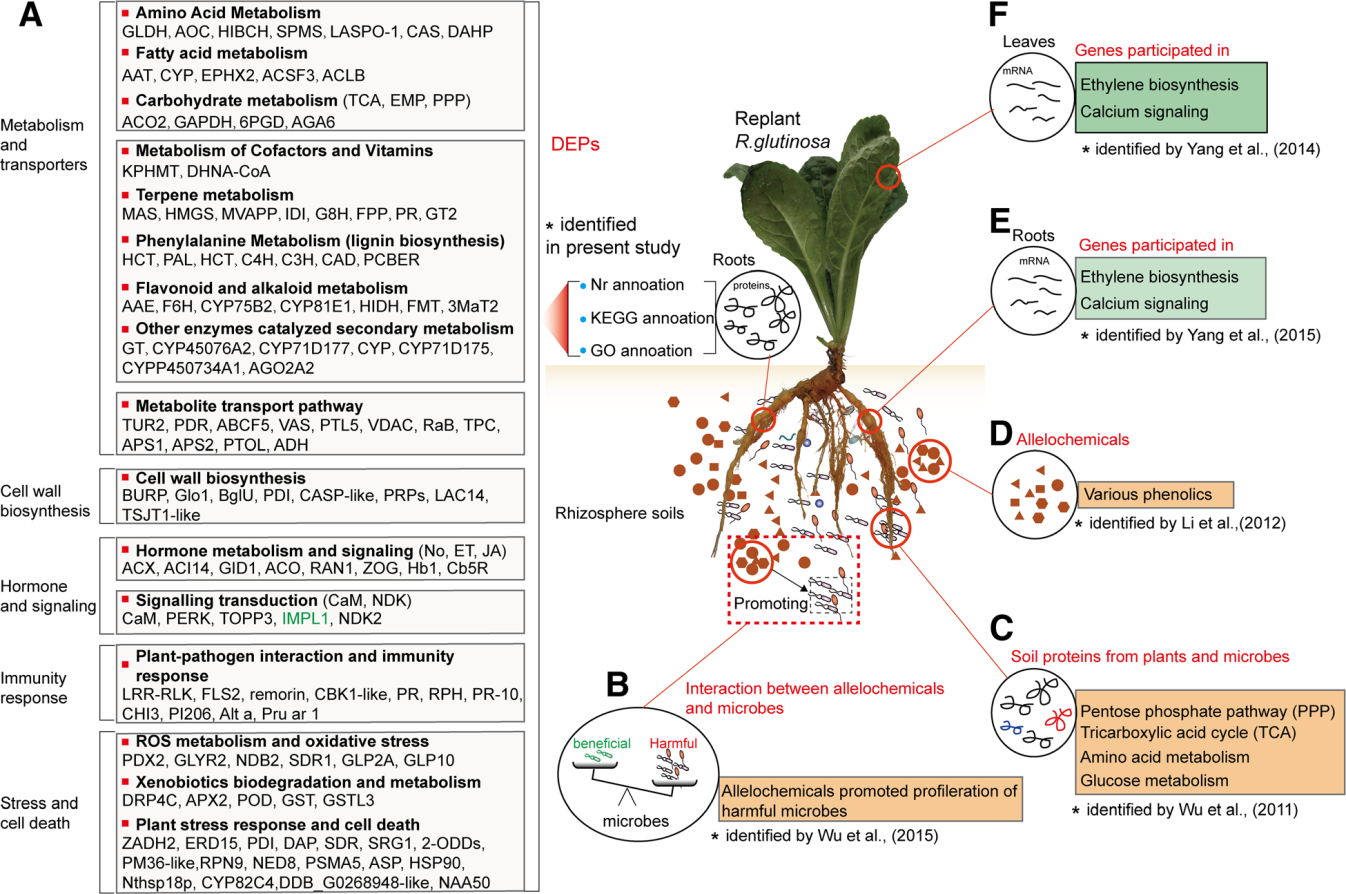
**a** DEPs in replanted *R. glutinosa* compared with normal-growth plants, and key molecular events caused by replant disease. Proteins listed in black were significantly upregulated in replanted *R. glutinosa*; those in blue were significantly upregulated in normal-growth *R. glutinosa*. **b**-**f** Comparison between the present study and previous studies conducted at different molecular levels. Some important molecular processes responding to replanting identified in this study were also found in previous studies. For example, proteins related to immunity systems were significantly upregulated in replanted *R. glutinosa* in the present study (**a**), reflecting a proliferation of microbes that might been mediated by phenolic allelochemicals (**b**) [11]. Some metabolic pathways (PPP and TCA) enhanced in replanted *R. glutinosa* (**a**) were observed in the rhizosphere of replanted *R. glutinosa* that was analysed by soil metaproteomics (**c**) [53]. The vital enzymes in phenylalanine metabolism (**a**), which generated various phenolic acids that have been identified as important allelochemicals (**d**) [12] were also highly expressed in replanted *R. glutinosa*. The ethylene and calcium signalling involved in sensing replant disease in the present study (**a**) were identified at the transcript level (**e**, **f**) [26, 27]

However, some DEPs had Nr annotations but failed to map to a GO slim in the GO database or to an EC entry in the KEGG database. Similarly, many transcripts that possessed GO slim and KEGG entries and an Nr annotation were not necessarily connected with “putative proteins”. To use DEPs comprehensively to obtain more functional annotations and determine corresponding molecular roles in replant disease formation, we integrated the Nr, Go and KEGG information and manually executed the classification for DEPs. For simplicity, only the prevalent role was considered when DEPs were involved in more than one biological process. The resulting 189 proteins were placed into 12 manually determined functional categories, including metabolic pathway, metabolite transport pathways, lignin biosynthesis, plant-pathogen interaction and immunity response, signal transduction, hormone metabolism and signalling, ROS metabolism and oxidative stress, xenobiotic biodegradation and metabolism, plant stress response and cell death, transcription factor, protein biosynthesis and other proteins. The complete list is provided in Additional file 11 and Fig. 5a.

**Fig. 6:**
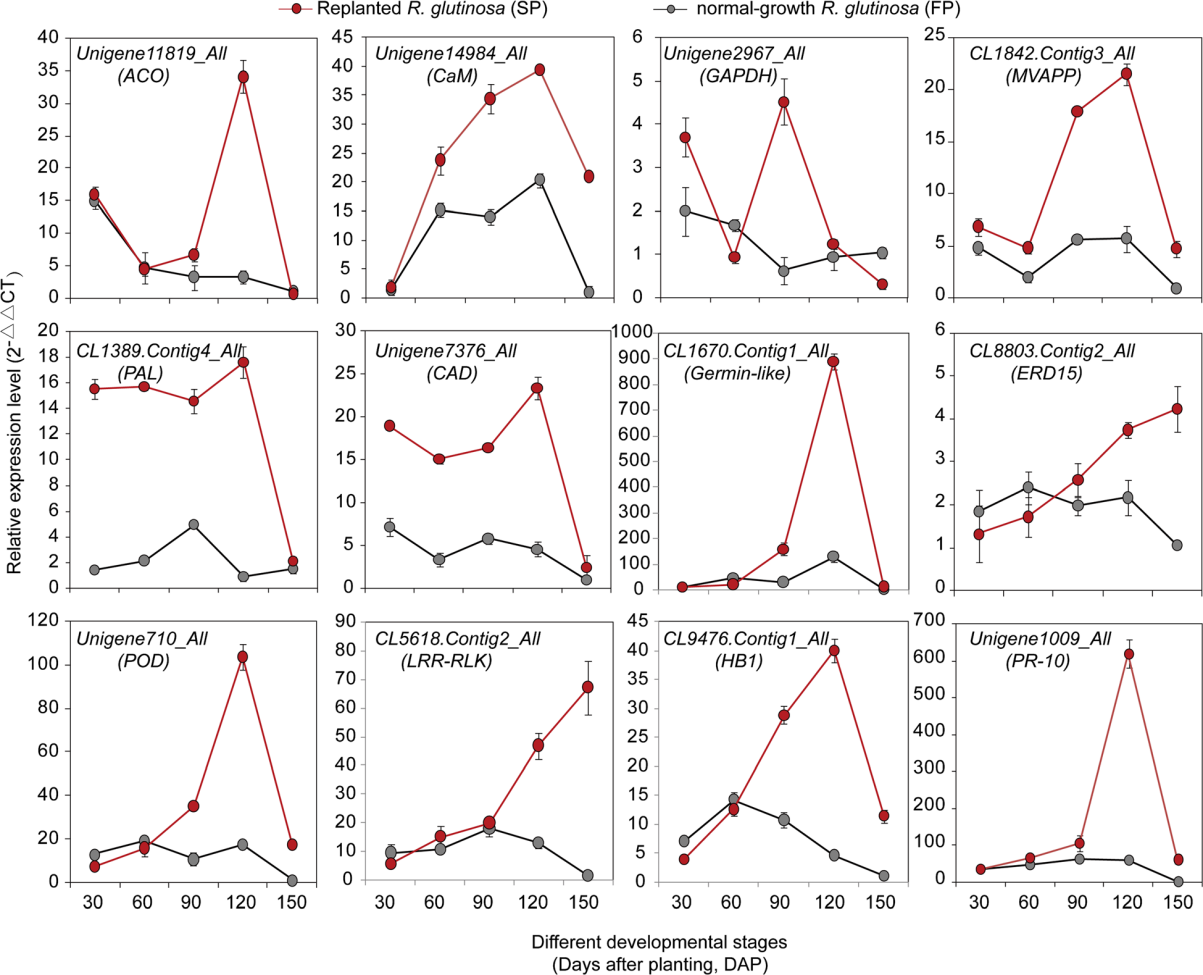
Expression profile of critical DEPs at the transcript level in replanted *R. glutinosa* compared with normal-growth plants during formation of replant disease. Error bars represent standard deviations (SD)

qRT-PCR was used to explore transcript changes during the development of normal-growth and replanted *R. glutinosa* (from 30 to 150 DAP) for 12 key genes encoding proteins involved in the above functional pathways. It was found that most of the genes were significantly upregulated at different stages in replanted *R. glutinosa* compared with normal-growth *R. glutinosa*, but the levels and abundances of genes at each stage were clearly different. *CaM* was expressed at high levels in replanted *R. glutinosa* throughout growth and development. Similarly, genes involved in hormone metabolism, immune systems and antioxidation, such as *ACO*, *HB1 (non-symbiotic haemoglobin class 1)*, *LRR-RLK*, *PR-10 (pathogenesis-related protein 10)*, *ERD15 (early responsive to dehydration 15)*, and *SOD*, had higher transcript levels in the last three stages (from 90 to 150 DAP). Of these genes, *ACO*, *HB1*, *germin-like*, *POD*, and *PR-10* had clear peaks in expression at 120 DAP in replanted *R. glutinosa*, but *ERD15* and *LRR-RLK* showed maximum expression at 150 DAP. Additionally, two genes that participate in lignin biosynthesis, *PAL* (*phenylalanine ammonia-lyase*) and CAD (*cinnamyl alcohol dehydrogenase*), were highly expressed in replanted *R. glutinosa* compared with normal-growth *R. glutinosa*, although the difference at 150 DAP was relatively lower. Simultaneously, of two metabolism-related genes, *MVAPP* (*mevalonate diphosphate decarboxylase*) was more abundant at 90, 120 and 150 DAP in replanted *R. glutinosa*, whereas *GAPDH* (*glyceraldehyde-3-phosphate dehydrogenase*) was more abundant at 30 and 150 DAP (Fig. 6).

## Discussion

The formation of replant disease is a complicated process that involves relationships among plants, autotoxic allelochemicals and microorganisms [5, 11, 22]. Clarification of the mechanism of how plants sense harmful signals generated by replanting is key for a deep understanding of the formation of replant disease. Previous studies have identified many genes that respond to replanting and have revealed some important molecular process related to replant disease formation at stages when replanted *R. glutinosa* is sensitive to injury [26, 27, 44]. However, these are not sufficient to fully explain the mechanism of replant disease formation at the single-transcript level. To identify more accurately the core harmful factors that result in replant disease formation at the protein level, this study generated an *R. glutinosa* transcriptome with full transcript information and constructed an *R. glutinosa* protein library. Based on this information, iTRAQ technology was used to identify 189 proteins that were significantly differentially expressed in replanted *R. glutinosa* compared with normal-growth plants at critical stages of replanting. Of these, 8 proteins were upregulated in normal-growth *R. glutinosa*. Most of these proteins were annotated as putative proteins, and some with obvious functional information indicated a relationship to *R. glutinosa* development. In contrast, 181 proteins were significantly upregulated in replanted *R. glutinosa*, demonstrating that *R. glutinosa* plants were severely injured by the practice of replanting. By the functional analysis of these proteins, some important molecular events that occurred in replanted *R. glutinosa* could be clearly observed.

### Replanting altered the metabolic balance of *R. glutinosa* and promoted efflux of potential allelopathic compounds

Three upregulated proteins in replanted *R. glutinosa*, ACO2, GAPDH and 6PGD (6-phosphogluconate dehydrogenase), were involved in glycolysis (EMP), the tricarboxylic acid (TCA) cycle and the pentose phosphate pathway (PPP), respectively, and an upregulated galactinol-sucrose galactosyltransferase 6 protein (AGA6) participated in the synthesis of raffinose. Simultaneously, six proteins related to amino acid metabolism and two DHAPs (3-deoxy-D-arabino-heptulosonate-7-phosphate synthase) that participate in the shikimate pathway were fully upregulated in replanted *R. glutinosa*. Additionally, five proteins associated with fatty acid synthesis were highly expressed in replanted *R. glutinosa* (Fig. 5a; Additional file 11). These results showed that replanting increased primary metabolism. The levels of enzymes involved in the synthesis of secondary metabolites increased, and six proteins involved in terpenoid biosynthesis, including HMGS (3-hydroxy-3-methylglutaryl-coenzyme A synthase), MVAPP, G8H (geraniol 8-hydroxylase), FPP (farnesyl diphosphate synthase), GT2 (Cinnamate beta-D-glucosyltransferase) and IDI (isopentenyl pyrophosphate isomerase), were much more abundant in replanted *R. glutinosa*. One protein, (+)-pulegone reductase, involved in the biosynthesis of monoterpenes were also more abundant in replanted *R. glutinosa* (Fig. 5a; Additional file 11). Terpenoids are a large class of medicinally active compounds [45]. However, in some studies, terpenoids had strong allelopathic potential in low concentrations [46]. In general, the biosynthesis of terpenoids is conducted primarily through the conserved MVA (mevalonate) and MEP (2-C-methyl-D-erythritol 4-phosphate) pathways in plants [47]. In this study, six of the proteins identified above were in the MVA pathway, but none was in the MEP pathway; therefore, replanting might promote the accumulation of terpenoids primarily via the MVA pathway. Furthermore, two unregulated proteins in replanted *R. glutinosa* were identified: momilactone A (MAS), which is a diterpenoid that might have been induced by a pathogen, and JA [48, 49], which has demonstrated potential allelopathic properties in rice [50].

Eight of the identified upregulated proteins participate in flavonoid metabolism and 10 in phenylalanine metabolism, and one (acetylajmalan acetylesterase) catalyses the final step in the biosynthesis of the indole alkaloid ajmaline (Fig. 5a; Additional file 11). Based on these results, metabolites related to carbohydrates, amino acids and fatty acids, sources of LMWs [7], had large accumulations in replanted *R. glutinosa* roots. Plant roots release many LMWs as exudates that significantly modify properties of the rhizosphere and change microbial communities [7]. These processes may significantly increase under abiotic and biotic stress by controlling activity levels of enzymes [51, 52]. The formation of replant disease occurs in a unique stressed environment; therefore, the metabolic balance of replanted *R. glutinosa* is disrupted and biased towards the generation of more exuded LMWs. Some studies suggest that the inhibition of plant growth that occurs with replanting leads to further increases in the efflux of exudates [53]. Many LMWs released from roots, such as fatty acids, long-chain fatty acids and amino acids, display potential allelopathic effects [53–55]. According to recent advances, some LMWs in *R. glutinosa* root exudates have specific roles in the regulation of rhizosphere microbial populations [56]. In this study, the highly expressed proteins in replanted *R. glutinosa*, as participants in LMW biosynthesis, were an indication that replanting increased the content of allelochemicals by promoting some key metabolic pathways. More importantly, these proteins were also found in the rhizosphere by metaproteomic methods [53]. Therefore, we hypothesize that with replanting of *R. glutinosa*, many of the compounds synthesized might be candidate autotoxic allelochemicals.

Through a series of transporters, accumulated allelochemicals in cells are ultimately released into the rhizosphere as root exudates. In this study, three proteins were identified as transporters, including one ABC transporter and two pleiotropic drug resistance proteins (TUR2 and PDR), which were highly expressed in replanted *R. glutinosa* (Fig. 5a; Additional file 11). ABC transporters transport diverse compounds in a variety of processes, which include the excretion of potentially toxic compounds, lipid translocation, nutrient transport and disease resistance [57]. TUR2 likely mediates the transport of toxic metabolites elicited by stress factors in plants [58], and PDR mediates the secretion of antimicrobial terpenoids from the plant surface into the rhizosphere [59]. One upregulated protein was the highly conserved PTL5 (polyol transporter 5), which transports a wide range of linear polyols, including sugars and amino acids [60, 61]. A voltage-dependent anion-selective channel (VDAC) and two VASs (lipid transfer proteins) were also upregulated in replanted *R. glutinosa*. VDACs transport many types of compounds, ranging from ions to large polymeric molecules. Plant VDACs are also involved in the PCD (programmed cell death) process, which is triggered by biotic and abiotic stresses [62]. VAS may facilitate the movement of lipids and are involved in the protection of plants against microbial infections [63]. These upregulated transporter proteins in replanted *R. glutinosa* display the typical properties of both transporter activity and pathogen defence, which are consistent with the typical characteristics of replant disease (replant stress and allelochemical secretion). We thus hypothesize that these proteins might transport metabolites from the roots into the rhizosphere under replant disease stress.

### Arrested tuberous root swelling in replanted *R. glutinosa* was mediated by lignin deposition

Plants use multiple strategies to defend against antagonistic pathogenic infection and adverse environmental effects, one of which is to strengthen cell walls with the deposition of lignin. In adverse environments, lignin deposition in cell walls builds effective barriers to protect plants from damage [64, 65]. In this study, we identified some key proteins, including two PAL, two HCTs (hydroxycinnamoyl-CoA shikimate/quinate hydroxycinnamoyl transferase), two C4Hs (cinnamic acid 4-hydroxylase), one cinnamate 4-hydroxylase (C3H) and two CADs involved in lignin synthesis, that were highly expressed in replanted *R. glutinosa* (Additional file 11). Phenylalanine is converted to coumaroyl-CoA by PAL, and coumaroyl-CoA is the first and most critical intermediate in the phenylpropanoid pathway. Cinnamic acid is hydroxylated by C4H to generate p-coumaric acid, which is immediately catalysed to form the corresponding CoA thioester by 4-coumarate-CoA ligase (4 coumaroyl-CoA synthase; 4CL). p-Coumarate 3-hydroxylase (C3H) is an important rate-limiting enzyme in monolignol biosynthesis. CAD catalyses the reduction of cinnamyl aldehyde to cinnamyl alcohol, and this alcohol is oxidized further to a lignin monomer. HCT transfers p-coumaroyl-CoA and caffeoyl-CoA to shikimate and quinate, respectively, which are used in the biosynthesis of the corresponding shikimate and quinate esters. HCT may significantly increase the lignin content. Upregulation of these proteins that catalyse lignin biosynthesis in replanted roots indicated that lignin accumulated primarily in the roots of replanted *R. glutinosa*. Many experiments have demonstrated that lignin deposited in cell walls gradually accumulates when plants encounter stressful environments [66, 67]. Replanting creates a unique stressed environment, in which many pathogens are induced by autotoxic allelochemicals. In our study, three PI206 proteins were highly expressed in replanted *R. glutinosa*, and PI206 is in the family of plant divergent proteins that are significantly induced by the fungal elicitor chitosan. Based on a previous study, in the biosynthesis of lignin, PI206 is used primarily to produce active lignin from two molecules of coniferyl alcohol [68]. Moreover, in recent reports, high levels of lignin in root cells significantly inhibited root growth of replanted plants [69–71]. Additionally, in some studies with different plants, allelochemicals significantly induced the biosynthesis of root cells.

The tuberous root is the important economic trait of *R. glutinosa*, with normal formation determining the product value. Most tuberous roots are derived from the swelling of adventitious roots, in which the continuous division of primary and secondary cambium cells in the stele is the primary driver to initiate tuberous root formation [72]. However, not all adventitious roots develop into tuberous roots. Physiological and molecular studies in plants with tubers or tuberous roots found that lignification of stele cells in fibrous roots prevents the formation of tuberous roots [73–75]. Therefore, tuberous root formation is a trade-off between two processes, and the balance between the proliferation of cambium cells and the deposition of lignin in stele cells determines whether adventitious roots will transform into tuberous roots. In the normal progression of tuberous root formation, lignin levels in cambium cells gradually decrease to facilitate cell division. For example, in a previous study, genes related to lignin biosynthesis were significantly inhibited in developing tuberous roots of *R. glutinosa* [72, 76]. However, based on the above data, replanting clearly promoted the synthesis of lignin by regulating different proteins. Thus, although lignin deposition in replanted *R. glutinosa* was a favourable behaviour to protect the plant, tuberous root formation was coincidentally inhibited. Additionally, phenylpropanoid and flavonoid metabolism are primarily pathways to generate phenolic acids, and the autotoxic allelochemicals identified in replanted *R. glutinosa* are phenolic acids. Therefore, upregulation of proteins related to the synthesis of phenylpropanoids and flavonoids demonstrated that phenolic acids were abundantly synthesized in replanted *R. glutinosa*. In addition, an induced stolon tip protein (TUB8) was expressed at low levels in replanted *R. glutinosa*. In potato, this protein is present at the early stages of tuberization and is related to tuber formation [77]. In plants, it is commonly associated with microtubule formation of specific organs [78]. Although TUB8 does not participate in the biosynthesis of lignin, its low expression in replanted *R. glutinosa* implied that replanting may have inhibited tuberous root formation of *R. glutinosa* in different ways, or essentially resulted from lignin in cambia cells.

### Immune defence systems were significantly induced in replanted *R. glutinosa* to resist a gradual increase in rhizosphere pathogens

In this study, two proteins identified as LRR-RLK kinases were highly expressed in replanted *R. glutinosa*. Two *R. glutinosa* pathogenesis-related 10 (PR10) proteins, which responded to replant disease in *R. glutinosa* in a previous study [56], were also significantly upregulated in replanting. Additionally, nine resistance-related proteins, including two PR proteins, one remorin protein, one chitinase 3, three PI206s and two allergen proteins, were highly expressed in replanted *R. glutinosa* (Fig. 5a; Additional file 11). These proteins are important components of plant immunity [79, 80]. The first line of defence is initiated by the detection of pathogen-associated molecular patterns (PAMPs) by plant cell surface pattern recognition receptors, primarily including LRR-RLK receptor proteins [81], which triggers a robust, PAMP-triggered immunity (PTI) defence response [82]. To circumvent the immune responses of plants, pathogens deliver effector proteins into plant cells to suppress PTI and facilitate pathogenesis [83]. As a countermeasure, plants use a second defence system in which NB-LRR protein-recognized, pathogen-derived effectors interact downstream with a series of PR proteins to initiate a hypersensitive response (HR). In comparison with PTI, ETI has greater specificity and efficiency in recognizing pathogens; thus, the function of PTI is to enable plants to survive the initial pathogen infection.

In this study, many proteins that function in plant ETI and PTI defence systems were activated in *R. glutinosa*, and their high levels of expression likely reflected the rapid proliferation of many pathogens in the rhizosphere of replanted *R. glutinosa*. In previous studies, the autotoxic allelochemicals secreted from *R. glutinosa* significantly altered the balance of microbial communities in the rhizosphere [84], and according to a recent advance, there is strong evidence that root exudates mediated the proliferation of pathogens to cause death of *R. glutinosa* in vivo [11]. When attacked by pathogens, the typical response of a plant is to initiate the immune defence system. Based on our results, the disease resistance system in replanted *R. glutinosa* was highly expressed, which was an indication that replanted *R. glutinosa* plants were under continuous attack by many pathogenic microbes. With the gradual accumulation of pathogens, the immune systems of *R. glutinosa* may finally be compromised, leading to death.

### Signalling pathway-mediated immune responses were activated in the roots of replanted *R. glutinosa*

**Fig. 7:**
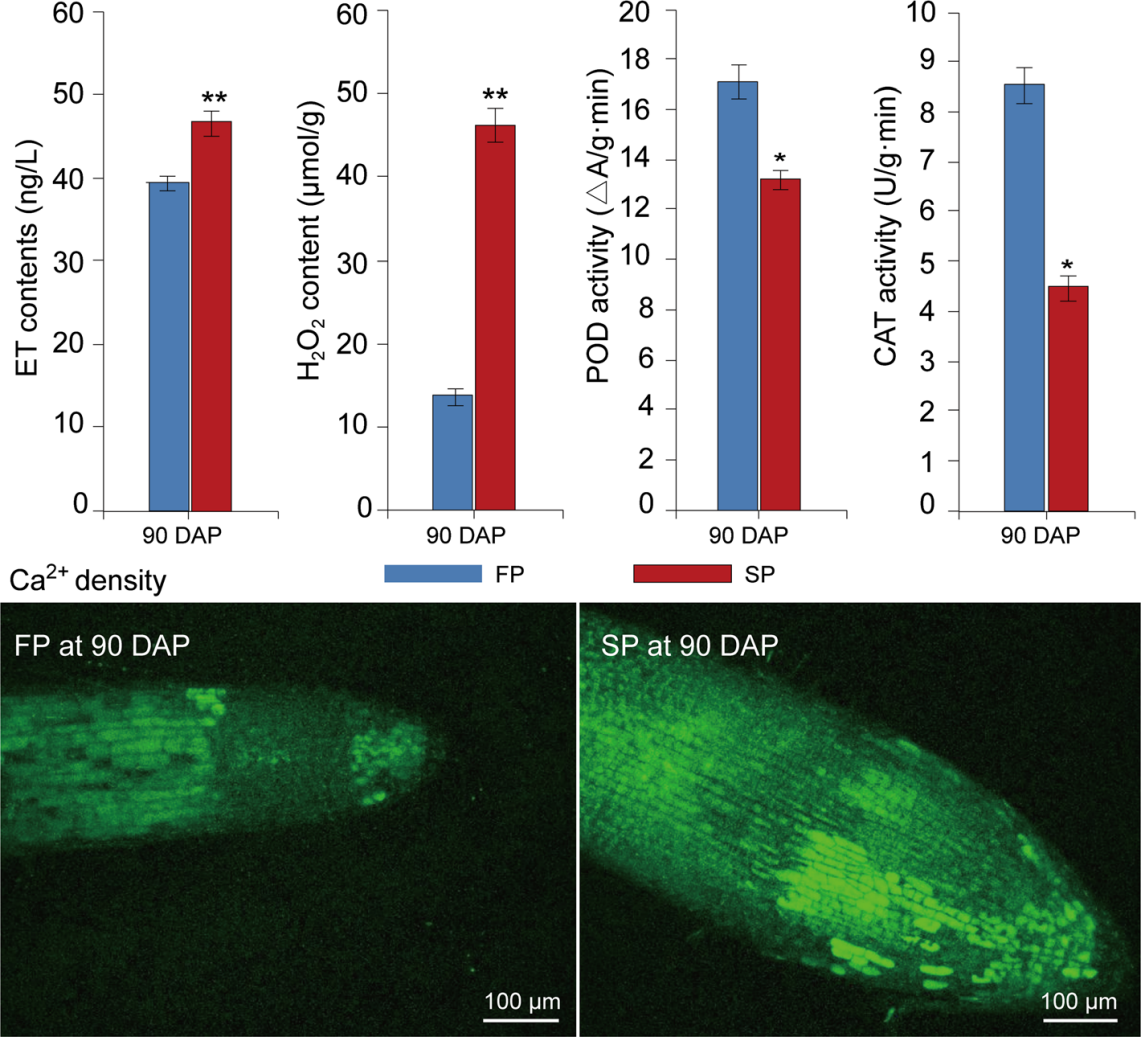
Differences in H_2_O_2_, Ca^2+^ density, and CAT and POD activities between replanted and normal-growth *R. glutinosa* at the most critical harmful stage caused by replanting (90 DAP). Error bars represent standard deviations (SD). * and ** represent significant differences of tested indexes between replanted and normal-growth *R. glutinosa* at *p* < 0.05 and *p* < 0.01, respectively, based on LSD test

In this study, 10 proteins involved in hormone metabolism and signalling were highly upregulated in replanted *R. glutinosa* compared with normal-growth plants (Fig. 5a; Additional file 11). Of these proteins, three were identified as ACO, a key enzyme in the synthesis of ET, and one as copper-transporting ATPase RAN1, which forms functional ethylene receptors in transgenic experiments [85]. In a previous study on transcript levels, the same result was observed, with many genes associated with ethylene metabolism and signalling highly expressed in replanted *R. glutinosa* [26, 27]. ELISA further confirmed that the ET content in replanted *R. glutinosa* was higher than in normal-growth plants (Fig. 7). Therefore, ET signalling was highly active in replanted *R. glutinosa*. Moreover, a protein was identified as non-symbiotic haemoglobin class 1 (HB1), which promotes the release of NO from mitochondria to the cytosol in response to hypoxia [86]. Furthermore, a protein identified as Acyl-CoA oxidase (ACX1A), which contributes to most of the JA production in wounded leaves [87], was highly upregulated in replanted *R. glutinosa*. Additionally, a gibberellin receptor protein, GID1, which participates in GA signalling, and a zeatin O-glucosyltransferase-like protein, which acts upon an inactive form of CK, were upregulated in replanted *R. glutinosa*. Overall, proteins closely related to ET, JA, and GA were significantly activated. In general, the biological functions of ethylene, JA, and GA are well characterized and widely involved in flower development, fruit ripening, senescence, and responses to biotic and abiotic stress. Increases in the biosynthesis of NO, ET and JA are associated with both PTI and ETI in plant resistance against bacterial infection, in which process these compounds can interact and regulate one another [88]. For example, ET is an elicitor of NO synthesis under stressful conditions, and zeatin, a prevalent cytokinin, and GA can reduce intracellular levels of NO [89]. Inactive CK and GID, with potential negative effects during CK and GA biosynthesis, were highly expressed, which indicated that NO activity was higher with replant stress*.* Based on these results, replant disease increased the production of NO, JA and ET, which regulated a series of downstream stress responses, such as HR and PCD.

In this study, a signal transduction protein, CaM, was also significantly expressed in replanted *R. glutinosa* (Fig. 5a; Additional file 11). Calcium signalling is an important bridge that links cellular responses to a wide variety of stress stimuli. In previous studies, calcium signalling participated in the formation of and response to replant disease in *R. glutinosa* [24–27]. Furthermore, the Ca^2+^ distribution in root cells of replanted and normal-growth *R. glutinosa* analysed using Fluo-3/AM indicated that the Ca^2+^ concentration was higher in replanted than in normal-growth *R. glutinosa* and confirmed that Ca^2+^ signalling participates in molecular responses of replanting (Fig. 7). Thus, we hypothesize that Ca^2+^ has essential roles in replant disease formation. Additionally, in different studies, Ca^2+^ signalling could regulate NO and ET production, and NO synthesis increases under stress conditions based on intracellular Ca^2+^ levels [89]. Different signalling pathways that form a complex regulatory network determine the outcome for replanted *R. glutinosa*.

### ROS levels and antioxidant enzymes were significantly induced in replanted *R. glutinosa*

In our studies, a series of proteins associated with ROS generation was upregulated in replanted plants. Germin-like oxalate oxidases and amine oxidases are a likely source of H_2_O_2_ in the apoplasts of plant cells [90]. Many studies have shown that increased plasma membrane NAD(P)H oxidase activity is associated with increased O_2_· − and H_2_O_2_ production under biotic and abiotic stresses [91]. ROS production in plants is accepted as a critical response caused by biotic and abiotic environmental stresses [92]. Additionally, ROS may act as signal molecules to trigger tolerance responses against various stresses [93]. In general, ROS accumulate in plant cells when stressors are encountered, and the activities of antioxidant enzymes such as SOD, POD and ascorbic acid peroxidase (APX) are rapidly altered to maintain resistance to this oxidative stress. Thus, ROS-generating and ROS-scavenging systems are in dynamic balance. However, with the excess production of ROS under continuous stress, ROS and ROS-scavenging systems are no longer in balance, and the corruption converts ROS into toxic molecules that damage cell macromolecules, inhibit cell growth and trigger cell death [22]. In this study, seven enzymes that function in the detoxification of toxic compounds were significantly induced in replanted *R. glutinosa*, including four GSTs, one ascorbate peroxide, one peroxidase and one DRP4C (dynamin-related protein 4C–like) (Fig. 5a; Additional file 11). Notably, an NDK protein was also significantly expressed in replanted *R. glutinosa.* In *Arabidopsis*, NDKs can interact with cytosolic catalases and play a key role in relieving oxidative stress [94]. These results suggested that roots of replanted *R. glutinosa* generated many ROS, and although a moderate level of ROS could be scavenged by the antioxidant system, replanted plants were in long-term stressed environments that ultimately overwhelmed the antioxidant system. In previous studies, replanting damaged leaves and root cells of *R. glutinosa* [10]; therefore, the activity of oxidative stress-related enzymes, including SOD, POD and CAT, among others, increased in replanted *R. glutinosa* to cause effects at the physiological level. However, compared with protein levels of SOD, POD and CAT at 90 DAP, their activities were significantly inhibited in replanted *R. glutinosa* at this point (Figs. 2a, 7). The consistency demonstrated that although SOD proteins were largely translated under replant disease stress, the physiological activities of these protein in plants were disturbed in replanted plants. Therefore, we hypothesize that the accumulation of ROS and loss of function of the scavenging system might be a critical factor in causing damage to replanted *R. glutinosa*.

### Long-term stress caused by consecutive monoculture ultimately led to the initiation and progression of plant cell death and senescence in *R. glutinosa*

In this study, two proteins involved in ER stress were highly abundant in replanted *R. glutinosa* (Fig. 5a; Additional file 11). One protein showed high homology with ERD15, which activates the osmotic and ER stress-induced cell death response [95]. The other protein was disulphide isomerase, which is associated with ER stress and apoptotic processes during prion infection in animals [96]. Stress conditions severely affect protein folding systems in the ER, which lead to accumulations of many misfolded or unfolded proteins [97], and these accumulations trigger cellular responses that include oxidative stress, calcium signalling and lipid generation to repair the cellular damage [98]. To eliminate the many abnormal proteins in the ER, the ER-associated degradation (ERAD) pathway efficiently recognizes and degrades these proteins, and in this process, the proteasome has an essential role [99]. In addition to participating in the ERAD pathway, the proteasome has important roles in controlling plant development and responses to stress [100]. In this study, five proteins were upregulated in replanted *R. glutinosa*: one RPN9, one PSMA5 (proteasome subunit alpha type 5), one NUB1L and two aspartic proteases, which were identified as proteasomes that might have multiple roles in replant disease. Ultimately, the result of ER stress is induction of PCD. Notably, RPN9 is associated with PCD, but simultaneously, the silencing of RPN9 elicited PCD in *N. benthamiana* [101]. Currently, the roles of proteasomes in the progression of PCD remain unclear. Ageing and death are often a synergistic process under long-term stress. In this study, one SRG protein was upregulated; this protein is closely linked with a senescence-related trait based on a QTL analysis and is highly induced in *Arabidopsis* during senescence [102]. Additionally, in our study, eight heat shock proteins (HSPs) were expressed abundantly in replanted *R. glutinosa*. HSPs and related chaperones act synergistically in response to stress factors to protect plants from cellular damage. HSPs are activated in response to a wide range of environmental stresses, including high and low temperatures, oxidative stress and osmotic stress. Moreover, HSP activity is significantly induced by different allelochemicals [103]. Based on these results, the stress of replanting significantly activated several stress-related processes, including ER stress, PCD and senescence, and the interaction of these processes likely accelerated the death of *R. glutinosa*. For replanted *R. glutinosa*, many factors might induce the progression of PCD; for example, the stress of pathogen attack and allelochemicals or the network formed by ETI, PTI and ROS.

### Integrating analysis of previous studies on different levels for replant disease of *R. glutinosa* revealed a mechanism for formation of replant disease

By analysing the above critical cellular process responding to replanting, we outlined the molecular process of replant disease formation. The present study found that proteins closely related to plant immune systems were significantly induced in replanted *R. glutinosa* (Fig. 5a). Previous studies have demonstrated that root exudates from *R. glutinosa* can significantly alter the microbial community of the rhizosphere, leading to relatively fewer beneficial microorganisms and more pathogenic microorganisms (Fig. 5b) [11, 84, 104]. Comparative analysis of two sets of results indicated the continuous proliferation of microbes in rhizosphere of replanted *R. glutinosa* mediated root exudates, inhibited plant growth and activated the immune system and defence response of replanted *R. glutinosa*. Simultaneously, we found that critical enzymes exuded from plants into the soil, including those involved in amino acid metabolism, the PPP and the TCA cycle (Fig. 5c) [53], were also upregulated in replanted *R. glutinosa* in the present study (Fig. 5a). This indicated that replanting altered the metabolic imbalance characteristic of replant disease, promoting metabolite efflux of replanted *R. glutinosa* towards the rhizosphere, which could further result in conversion to allelochemicals (Fig. 5d) [4, 12]. Notably, key enzymes catalysing lignin metabolism also significantly increased in replanted *R. glutinosa* roots (Fig. 5a). By coincidence, the accumulation of lignin can hider division of cambium cells in tuberous roots, which provides a critical clue to explain how tuberous root formation is inhibited under the practice of consecutive monoculture.

**Fig. 8:**
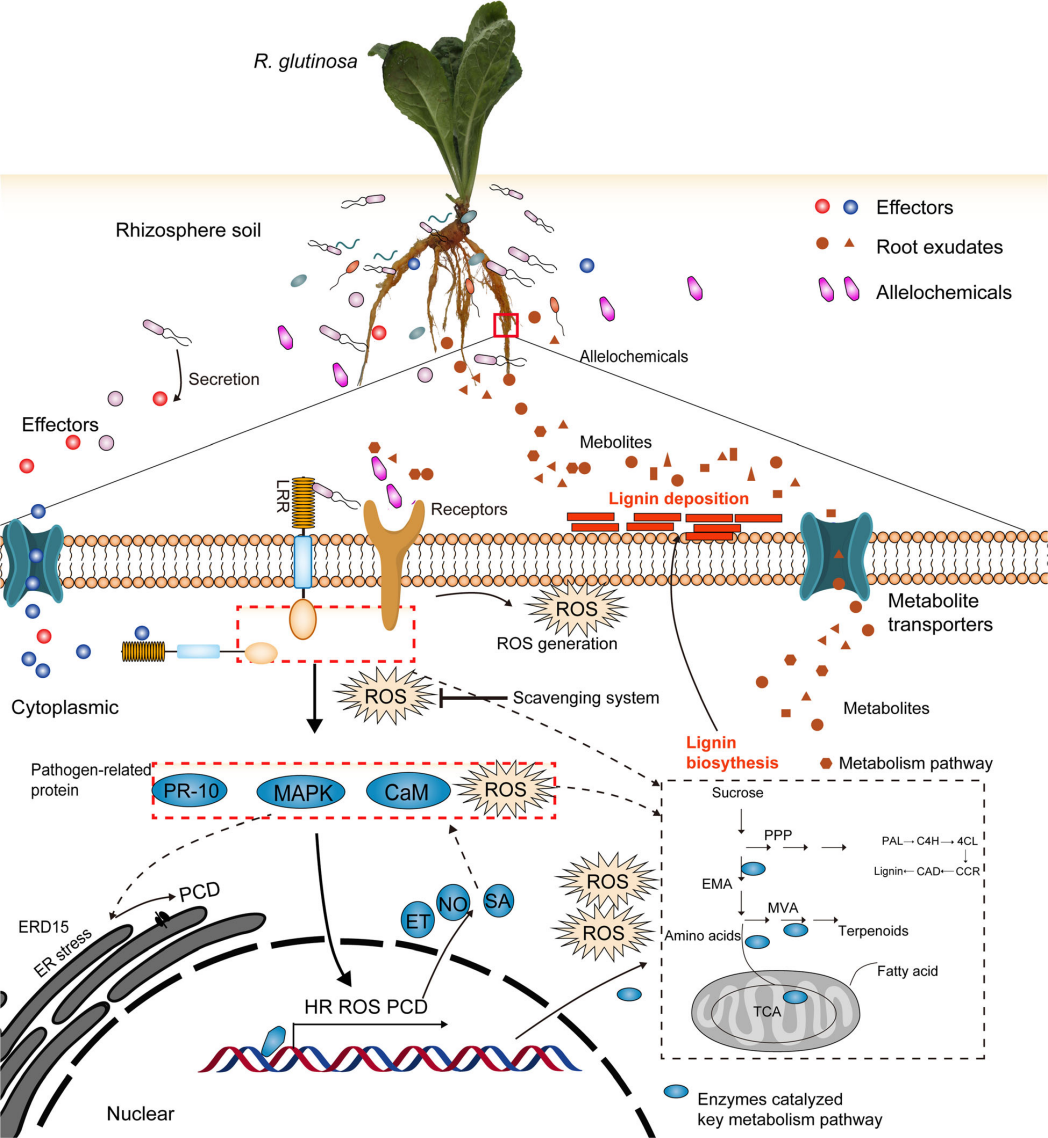
Hypothetical model for the mechanisms causing damage in replant disease of *R. glutinosa*

Furthermore, we found that crucial proteins participating in ET metabolism and signalling and calcium signalling identified from transcript data (Fig. 5e,f) [26, 27, 44] were also found to be upregulated in replanted *R. glutinosa* plants in the present study (Fig. 5a). Many reports have shown that allelochemicals can singly activated ET, JA and Ca^2+^ signalling pathways, promote ROS accumulation and change metabolic balance in different plants [24, 25, 105]. However, different studies also indicated that the immune response can trigger a series of downstream events, including ROS generation, PCD, and ER stress, among others [106–108], which have been clearly identified in this study (Fig. 5a). We thus hypothesize that these signalling and stress processes might be activated by either immune systems mediated by pathogens or stress signals induced by allelochemicals, both of which were present in the rhizosphere of replanted *R. glutinosa*, more likely by their interaction. Regardless of the source of harmful signals, these processes had significant negative effects on plant growth and development, and the interaction of these events ultimately resulted in the formation of replant disease. Based on the integrated analyses of these events in different studies, one possible scenario for the harmful mechanisms of replanting in *R. glutinosa* is illustrated in Fig. 8, although the details remain to be confirmed.

## Conclusions

*R. glutinosa* is one of the main medicinal plants that suffers from replant disease. This study constructed and integrated a high-capacity *R. glutinosa* transcriptome, and on this basis, a special *R. glutinosa* proteome library was obtained. This information fills the gap of genetic information in *R. glutinosa* and thus provides important information for further in-depth research on the molecular mechanism of replant disease formation, the mechanisms of growth and development of *R. glutinosa* and the biosynthetic mechanisms of the medicinal active ingredients. More importantly, based on the proteome library, the critical proteins responding to replanting were clearly identified. By analysis of these proteins and previous studies, we propose a mechanism for the damaging effects of replant disease, in which major signalling pathways transmit a harmful signal from the rhizosphere in replanted plants, followed by activation of a series of downstream cellular death processes (Fig. 8). These findings deepen the understanding of the molecular mechanisms of replant disease formation and provide a theoretical basis for solving the problem of replant disease in Chinese medical material production.

## Additional files

Additional file 1: Primer sequences for the 12 key genes that were differently expressed at the protein level in replanted *R. glutinosa* compared with normal-growth ones. (XLSX 9 kb)

Additional file 2: Summary of assembly results for different transcriptome libraries of *R. glutinosa*. (XLSX 9 kb)

Additional file 3: Full unigene length distribution of the *R. glutinosa* transcriptome. (DOCX 77 kb)

Additional file 4: Length distribution of proteins translated from the full *R. glutinosa* unigene set. (DOC 351 kb)

Additional file 5: Annotation statistics of *R. glutinosa* full unigenes in different databases. (XLSX 8 kb)

Additional file 6: GO annotation of *R. glutinosa* full-transcriptome sequences. (DOC 288 kb)

Additional file 7: COG annotation of *R. glutinosa* full-transcriptome sequences. (DOC 151 kb)

Additional file 8: Detailed KEGG pathways of all unigenes from the *R. glutinosa* transcriptome. (XLSX 14 kb)

Additional file 9: Unique peptide sequences identified in the present study. (XLSX 2281 kb)

Additional file 10: Identification of all detected proteins in the present study. (XLSX 493 kb)

Additional file 11: Differentially expressed proteins in replanted *R. glutinosa* compared with normal-growth *R. glutinosa*. (XLSX 111 kb)

Additional file 12: KEGG pathway analysis of differentially expressed proteins (DEPs) in replanted *R. glutinosa* compared with normal-growth plants. (XLSX 19 kb)

## Abbreviations

6PGD: 6-phosphogluconate dehydrogenase
CAD: Cinnamyl alcohol dehydrogenase
DAP: Days after planting
DEPs: Differentially expressed proteins
EMP: Embden–Meyerhof–Parnas
ERD15: Early responsive to dehydration
FDR: False discovery rate
GAPDH: Glyceraldehyde 3-phosphate dehydrogenase
HB: Non-symbiotic haemoglobin class 1
iTRAQ: Isobaric tag for relative and absolute quantification
MVAPP: Mevalonate diphosphate decarboxylase
PAL: Phenylalanine ammonia-lyase
PPP: Pentose phosphate pathway
PR-10: Pathogenesis-related protein 10
ROS: Reactive oxygen species
SOD: Superoxide dismutase
TCA: Tricarboxylic acid cycle
TTC: Triphenyl tetrazolium chloride
