# Supplementary material for: Differential proteomic analysis of replanted Rehmannia glutinosa roots by iTRAQ reveals molecular mechanisms for formation of replant disease
Source: BMC Plant Biol. 2017 Jul 10;17:116. doi: 10.1186/s12870-017-1060-0 (PMC5504617; doi:10.1186/s12870-017-1060-0)
Supplement: Supplementary file 3 — Full unigene length distribution of the R. glutinosa transcriptome. (DOCX 77 kb) [file 12870_2017_1060_MOESM3_ESM.docx]

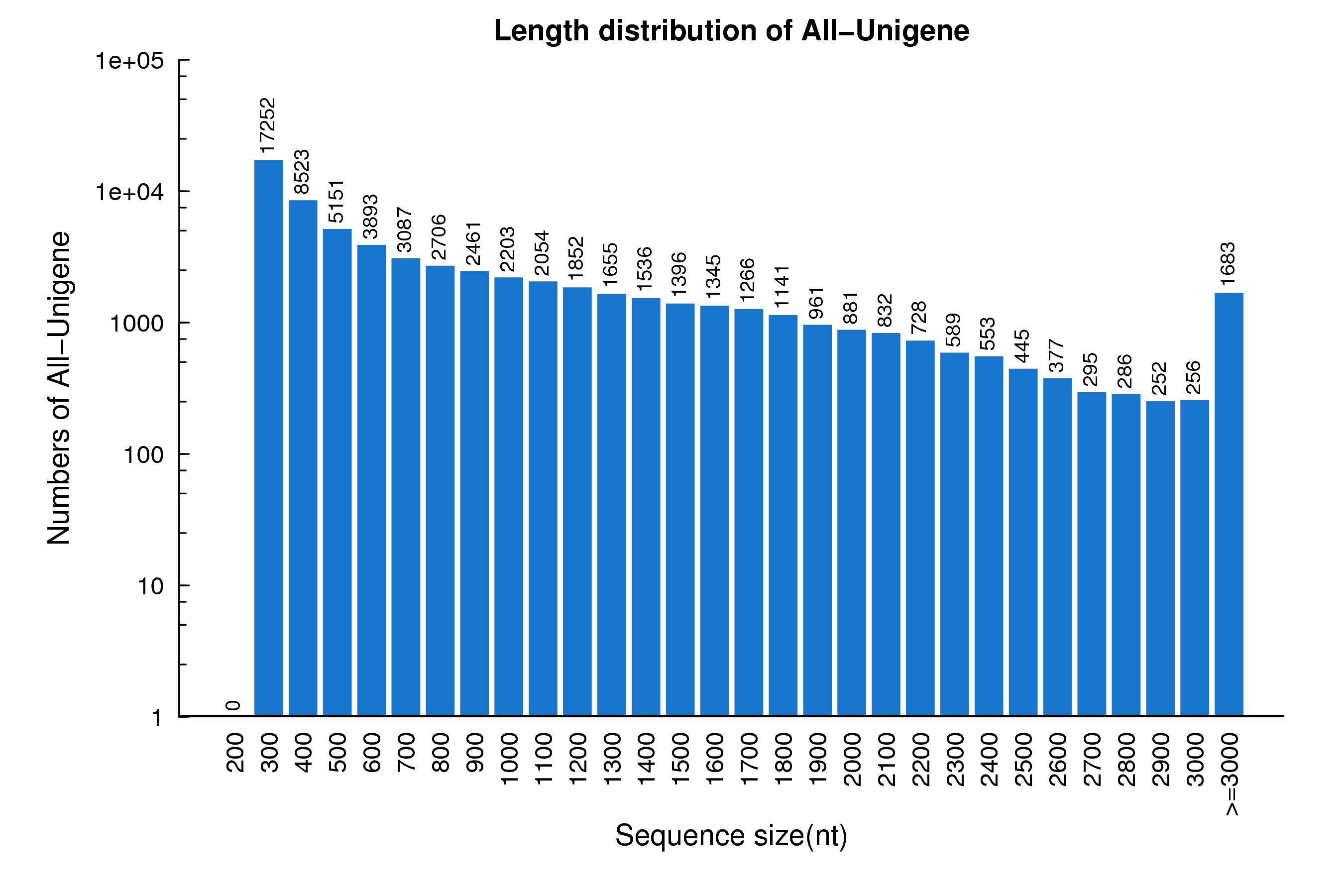


**Additional file 3. Full unigene length distribution of *R. glutinosa* transcripome that assembled from different library sequences.**
