# Supplementary material for: Differential proteomic analysis of replanted Rehmannia glutinosa roots by iTRAQ reveals molecular mechanisms for formation of replant disease
Source: BMC Plant Biol. 2017 Jul 10;17:116. doi: 10.1186/s12870-017-1060-0 (PMC5504617; doi:10.1186/s12870-017-1060-0)
Supplement: Supplementary file 4 — Length distribution of proteins translated from the full R. glutinosa unigene set. (DOC 351 kb) [file 12870_2017_1060_MOESM4_ESM.doc]

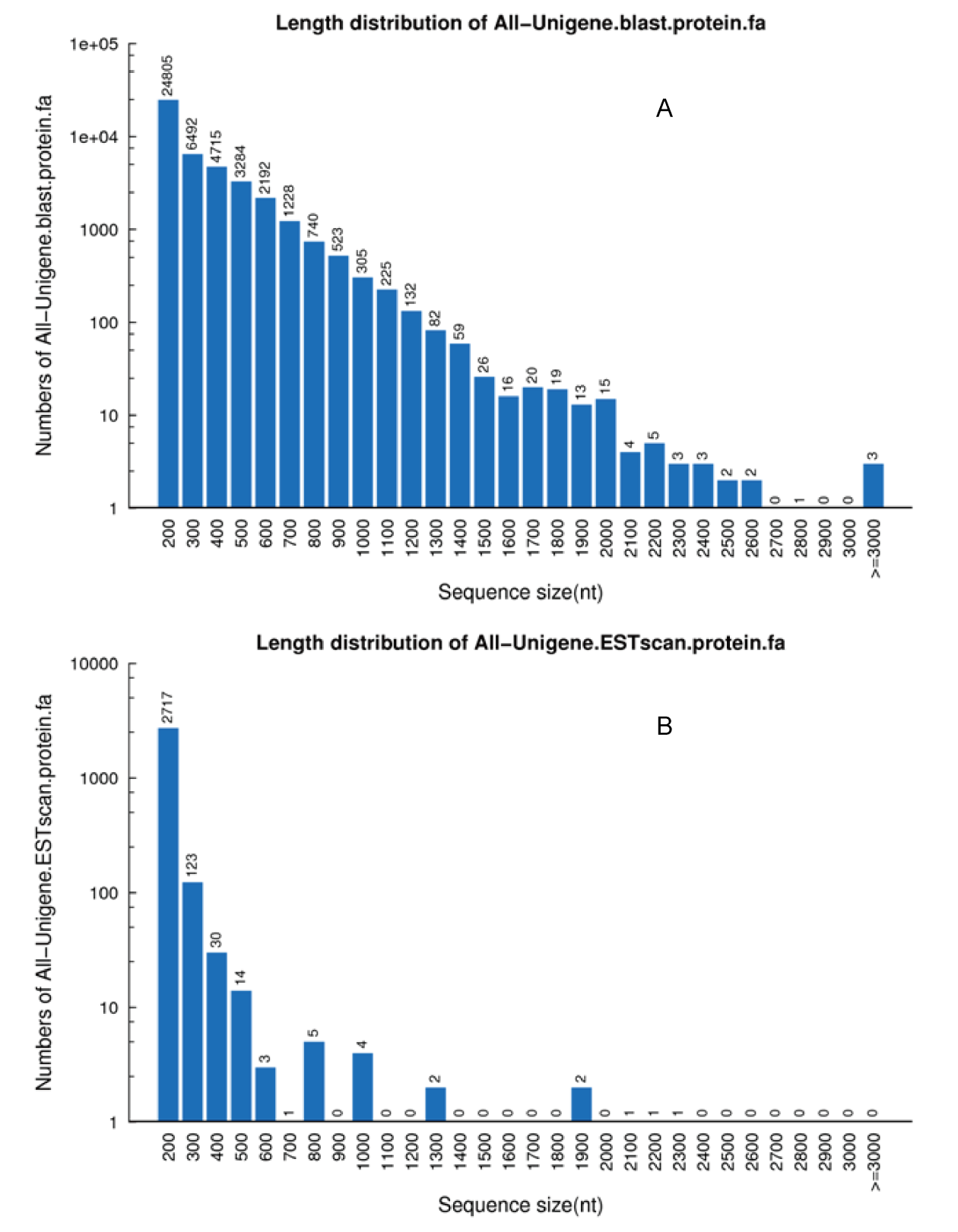


**Additional file 4. Length distribution of proteins translated from full *R. glutinosa* unigenes, based on (A) BlastX and (B) ESTscan.**
