# Supplementary figures and images for: Differential proteomic analysis of replanted Rehmannia glutinosa roots by iTRAQ reveals molecular mechanisms for formation of replant disease
Source: BMC Plant Biol. 2017 Jul 10;17:116. doi: 10.1186/s12870-017-1060-0 (PMC5504617; doi:10.1186/s12870-017-1060-0)

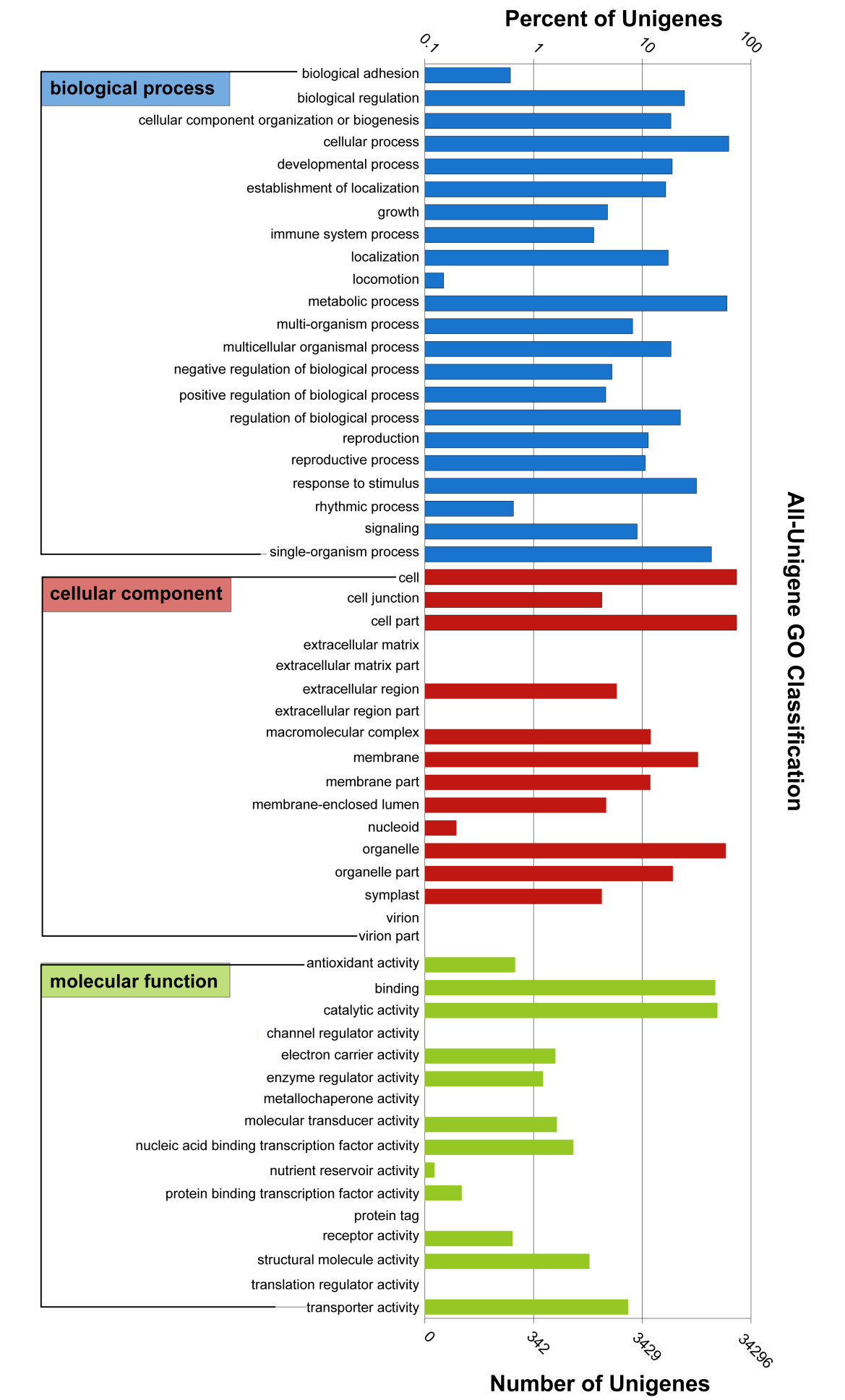


**Additional file 6. GO annotation of *R. glutinosa* full transcriptome sequences.**

Supplement: Supplementary file 6 — GO annotation of R. glutinosa full-transcriptome sequences. (DOC 288 kb) [file 12870_2017_1060_MOESM6_ESM.doc]

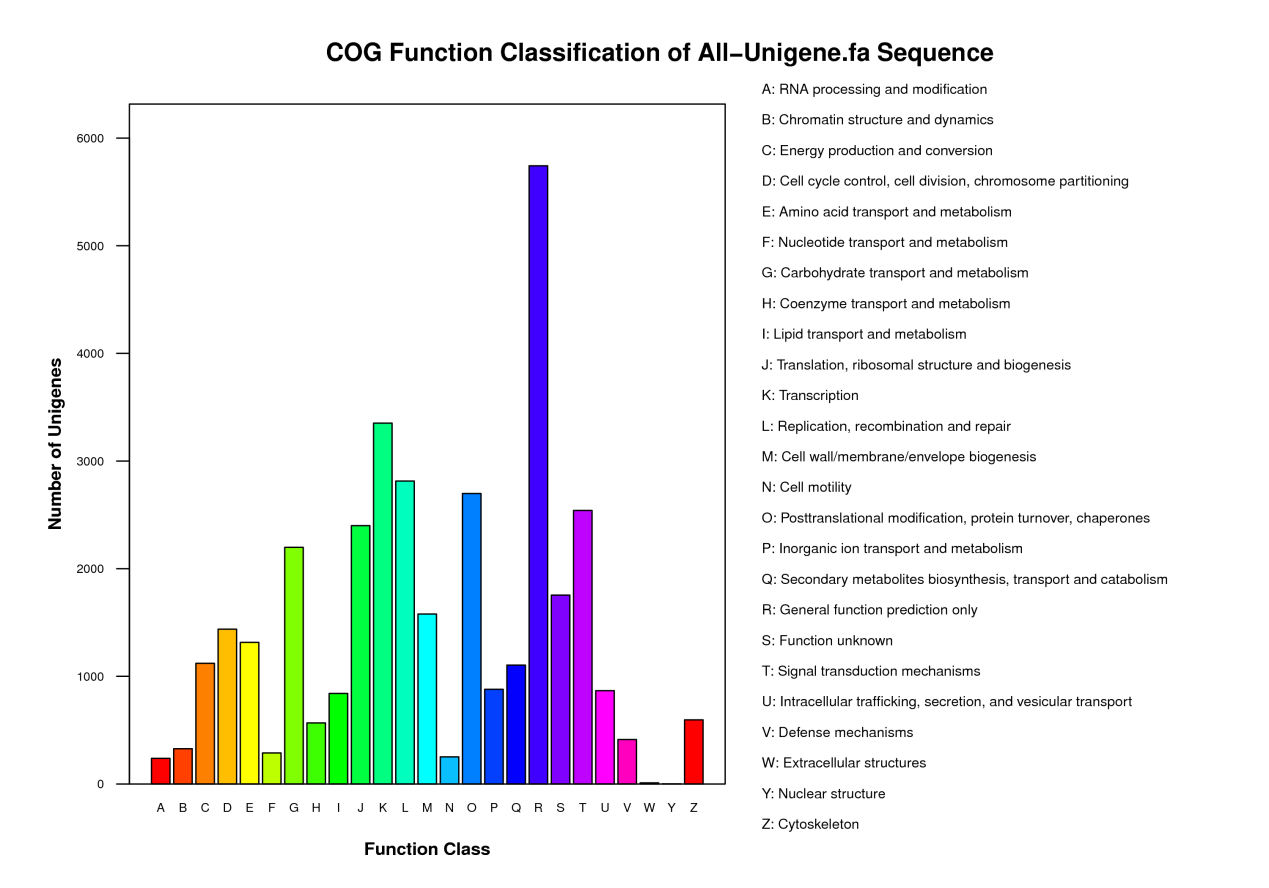


**Additional file 7. COG annotation of *R. glutinosa* full transcriptome sequences.**

Supplement: Supplementary file 7 — COG annotation of R. glutinosa full-transcriptome sequences. (DOC 151 kb) [file 12870_2017_1060_MOESM7_ESM.doc]
